# Kinetic Traps in RNA Folding: Targeted Design of Frameshifting Element Mutants by Thermodynamic and Kinetic Analysis of the Chikungunya Virus

**DOI:** 10.1021/acs.jpcb.5c08223

**Published:** 2026-01-29

**Authors:** Samuel Lee, Tamar Schlick

**Affiliations:** † University of Pennsylvania, Philadelphia, Pennsylvania 19104, United States; ‡ Department of Chemistry, 5894 New York University, New York, New York 10003, United States; § Courant Institute of Mathematics, Computing, and Data Science, 5894 New York University, New York, New York 10012, United States; ∥ NYU-ECNU Center for Computational Chemistry, NYU Shanghai, Shanghai 200062, PR China; ⊥ NYU Simons Center for Computational Physical Chemistry, 5894 New York University, New York, New York 10003, United States

## Abstract

Chikungunya virus (CHIKV) employs a programmed ribosomal frameshifting element (FSE) to regulate the synthesis of its viral proteins, making the FSE an attractive antiviral target. Yet the structural dynamics that govern its function are complex and poorly understood, with multiple folds discovered. Through computational analysis, we suggest that the FSE’s conformation is determined by a competition between thermodynamic stability and cotranslational folding kinetics. Using an integrated computational pipeline, we map the FSE’s equilibrium landscape, revealing a thermodynamically favored pseudoknot that emerges only with sufficient flanking residues. We then use kinetic simulations to show that, for the wildtype sequence, this pseudoknot is often kinetically trapped in simpler, less stable stem loop structures that form more rapidly during synthesis. Using this information, we rationally design several mutants to target different folds in the FSE’s repertoire. We demonstrate that while a purely thermodynamic design can fail due to kinetic traps, an iterative design procedure, informed by kinetic analysis, can drive the FSE onto a target conformation. Our work explores conformational plasticity and multiple folding pathways of the CHIKV FSE, shows how cotranslational kinetics influence the fold-switching landscapes, establishes a computational framework for kinetic-based RNA engineering, and highlights the importance of considering folding pathways in the design of RNA-targeted therapeutics.

## Graphical abstract

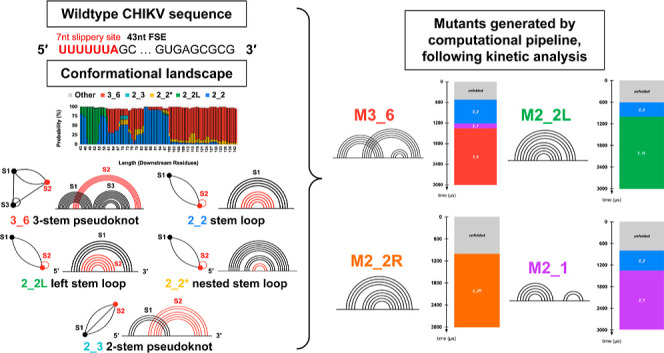

## Introduction

Chikungunya virus (CHIKV) is a mosquito-borne alphavirus that poses a major public health threat due to its ability to cause severe arthralgia (joint stiffness), which can significantly reduce the quality of life. The primary vector of CHIKV is the *Aedes* mosquito, which thrives in tropical and subtropical climates, facilitating rapid spread in densely populated regions. The re-emergence of CHIKV and its increasing incidence globally, particularly in previously unaffected areas like parts of Europe and the Americas, have spurred renewed virological and epidemiological interest.^1,2^

As a positive-sense RNA virus, CHIKV relies on intricate interactions between its genomic RNA and host cell machinery throughout its life cycle, including transcription, translation, and genome packaging.^3^ A central determinant of these processes is the secondary structure of the viral RNA, which can modulate ribosomal pausing, translation efficiency, and the recruitment of host factors. However, the dynamic interplay between equilibrium folding landscapes and cotranslational kinetics in CHIKV remains poorly understood.^4,5^

Among these regulatory structures, programmed −1 ribosomal frameshifting (−1 PRF) is a critical translational mechanism that allows CHIKV to expand its protein repertoire. This event is triggered at a conserved slippery sequence (UUUUUUA) within the 6K coding region and is stimulated by a downstream frameshifting element (FSE), a 36-nucleotide segment capable of adopting multiple conformations—ranging from H-type pseudoknots to simple stem loops—that regulate synthesis of the transframe (TF) protein (Figure 1).^6^ Disrupting −1 PRF has been shown to impair TF production and attenuate viral propagation, positioning the FSE as an attractive antiviral target.^7,8^

**Figure 1:**
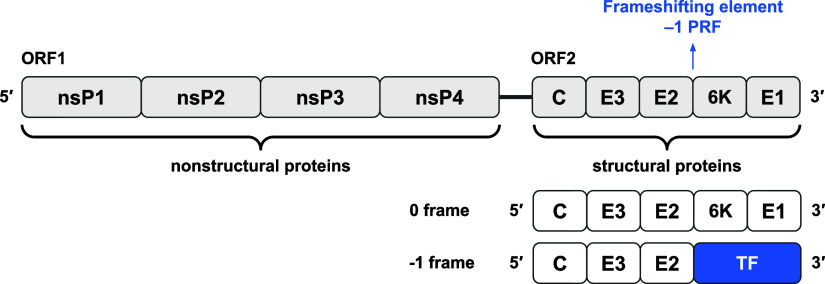
Genome organization of CHIKV. The CHIKV genome consists of two open reading frames (ORFs). ORF1 encodes nonstructural proteins (nsP1 nsP4) essential for replication, while ORF2, transcribed from 26S subgenomic RNA (sgRNA), encodes structural proteins (C, E3, E2, 6K, and E1). A programmed −1 ribosomal frameshift (−1 PRF) occurs at a slippery site with a downstream frameshifting element (FSE), producing an alternative polyprotein with a TF domain instead of 6K.

Many structural and functional studies for CHIKV as shown in Table 1 have shown that the CHIKV FSE adopts dynamic structures capable of regulating −1 PRF, with SHAPE-MaP and ribosome profiling data supporting both stem loop and pseudoknot conformations.^8–10^ However, reported structures vary widely by method and context: while Hanson et al. and Kendra et al. highlight a functional pseudoknot near the 6K region, others describe alternative folds including kissing loops, bulged stems, or stem loop intermediates.^11,12^ Computational studies have likewise offered various models, with divergent predictions of which FSE structures dominate under physiological conditions.^13,14^ These works raise two central questions: (1) which specific RNA topologies are thermodynamically versus kinetically favored across the CHIKV lifecycle, and (2) whether small sequence changes can bias the folding pathway in a predictable, motif-specific manner. Relevant RNA topologies for the CHIKV FSE folds are shown in Figure 2, including a 3-stem pseudoknot and stem loops. We label motifs with graph notation using RNA-As-Graphs (RAG), which are broader than 2D structures and which we use in this study to annotate conformational landscapes (see Materials and Methods).

**Table 1:** Summary of CHIKV FSE Studies^a^

study	scope/region	technique	proposed structure	key finding
Hanson et al. (2024)^10^	∼1.5 kb region flanking the FSE	SHAPE-MaP & RNA probing	pseudoknot & stem loop	conserved pseudoknot and stem loop structures regulate frameshifting.
Diaz (2024)^11^	250–350 nt capsid-E3 junction	RNA structure mapping	kissing-loop	kissing-loop interactions identified in the FSE.
Tants and Schlundt (2024)^12^	∼40–80 nt alphavirus FSEs	NMR spectroscopy	bulged stem loop	bulged region in the FSE stem loop is essential for PRF.
Madden et al. (2021)^9^	CHIKV TF protein expression	ribosome profiling	*functional*	FSE-dependent ribosomal pausing and TF synthesis confirmed.
Nagy & Alhatlani (2021)^14^	*In silico* RNA models	molecular dynamics simulations	stem loop	FSE stem loop stability and transition dynamics predicted.
Zimmer et al. (2020)^13^	CHIKV FSE (in silico)	RNA folding analysis	pseudoknot	dynamic pseudoknot formation is a key regulator of PRF.
Kendra et al. (2018)^8^	CHIKV 6K coding region	molecular modeling & SHAPE-MaP	pseudoknot	RNA secondary structure regulates ribosomal pausing and efficiency.
Firth and Atkins (2008)^32^	CHIKV, SFV, SINV FSEs	sequence alignment and conservation mapping	pseudoknot	conserved, pseudoknotted PRF elements in alphaviruses identified.

a An overview of key studies analyzing the CHIKV FSE. This summary highlights the different techniques used, the regions studied, and the often-conflicting structural and functional findings that motivate the present work. The entry “*functional*” refers to studies that confirmed the biological function or activity of the frameshifting region without determining its precise fold.

**Figure 2:**
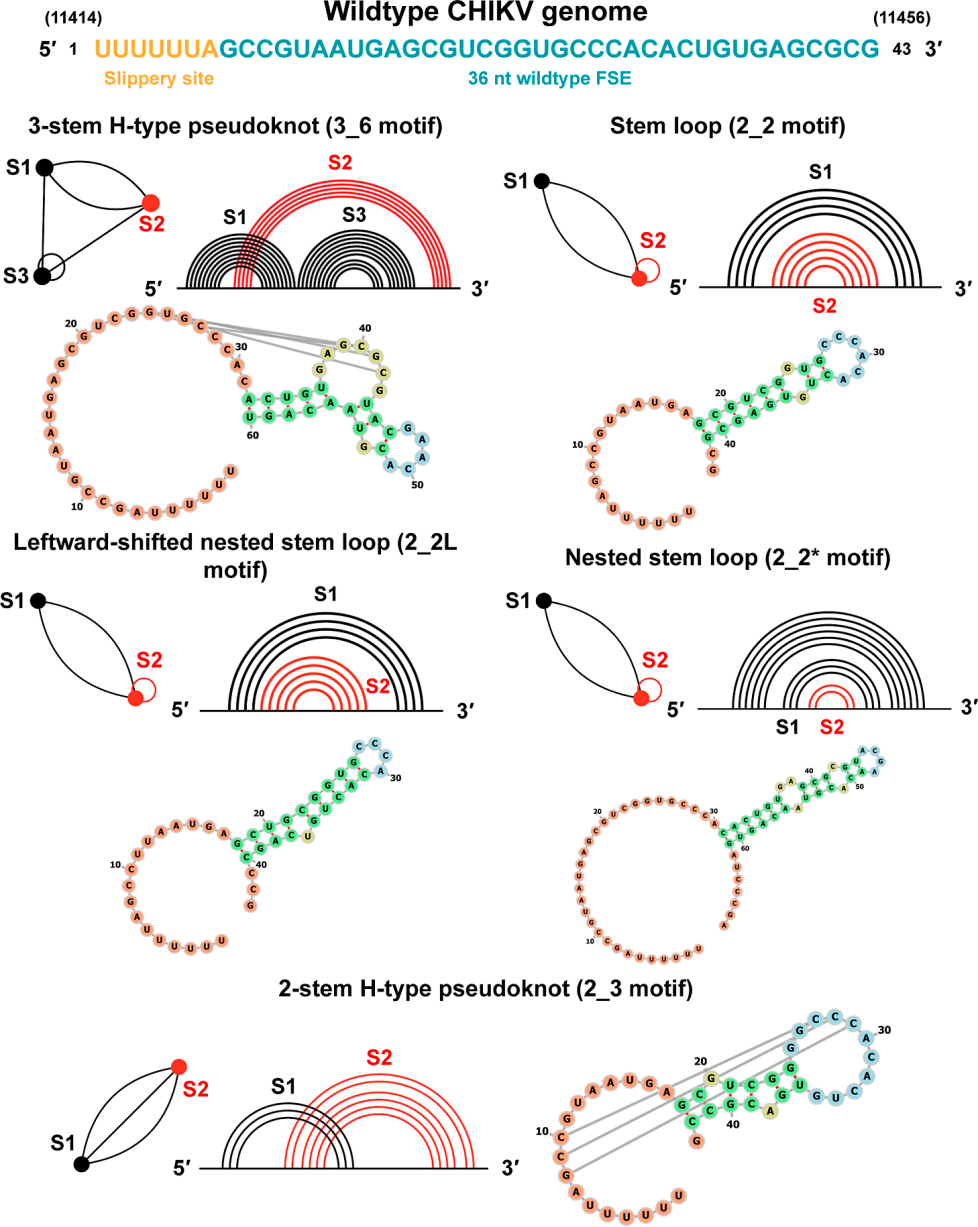
Structural motifs in the wildtype Chikungunya virus (CHIKV) frameshift-stimulating element (FSE). The CHIKV genome segment (positions 11414–11457) encompassing the slippery site (orange) and wildtype FSE region (blue) is illustrated, showing different predicted RNA secondary structures: a 3-stem H-type pseudoknot (3_6 motif), a simple stem loop (2_2 motif), a leftward-shifted nested stem loop (2_2L motif), a nested stem loop structure (2_2* motif), and a 2-stem H-type pseudoknot (2_3 motif). Each RNA structural motif is represented using two visualization schemes: arc diagrams depicting nucleotide base-pairing (right) and dual graphs (left). In the dual graphs, stems are represented as vertices (•), and loops/bulges are edges (•—•).

More generally, much research has focused on better understanding the frameshifting mechanism and the relation between the stability of the FSE and the frameshifting efficiency.^15–17^ The process is complex because multiple ribosomes are involved and, hence, continuous refolding and unfolding occurs. This means that different folds for the FSEs can form, with varying stabilities and frameshifting efficiencies, depending on the ribosome-occupied and ribosome-accessible nucleotides. For this reason, conformational plasticity of the FSE appears important, so various intermediates can refold into a stable FSE structure. Tension generated in the spacer region also appears important in the FSE folding pathway and associated transitions.^18^

To study these complex kinetic, thermodynamic, and functional aspects of −1 PRF, many experimental studies have employed single molecule techniques, SHAPE and DMS chemical mapping, in vitro functional measurement, and others to probe these aspects in viral systems. For example, force microscopy studies of the West Nile Virus (WNV), known for very high frameshifting efficiency (up to 80%), revealed complex and multiple dynamic pathways (involving different structures, pseudoknots, and double stem loops) that are influenced by the tension generated by ribosomes.^19^

Similarly, in their kinetic analysis of the formation of a frameshifting RNA pseudoknot by optical tweezer experiments complemented by molecular dynamics analysis, Hsu et al.^20^ emphasized the importance of conformational plasticity by arguing that successive translating ribosomes mediate the folding and refolding of the pseudoknot. This structure coordination is important to rescue trapped folding intermediates and lead them to highly stable structures. Folding and unfolding pathways are frequent as the landscape of nucleotides occupied by ribosome evolves.

Importantly, the folding intermediates and structures involved in FSE translation also depend on the upstream and downstream nucleotides. For SARS-CoV-2, it was shown that both upstream and downstream nucleotides may compete to define the stimulatory element.^16,21^ Pekarek et al. showed the complex interaction between upstream and downstream residues (5′ and 3′ of the FSE) in forming various pseudoknots and stem loops by single-molecule techniques. In Lee et al., we explored these conformational landscapes computationally to propose sequential transitions during multiple ribosome translation.

These works and others^22–24^ clearly emphasize the complex interplay between kinetic and thermodynamic stability of the FSE. They also raise new ideas for designing mutant FSEs by altering or suppressing the involvement of certain nucleotides to generate the desired FSE folding outcome.

Indeed, recent chemical probing and mutational techniques have manipulated the FSE using SHAPE-MaP, DMS mapping, and targeted mutagenesis to demonstrate that mutations within the frameshifting element can destabilize key stems or tertiary contacts, weakening ribosomal pausing and shifting translation outcomes.^6,25^ For example, Bhatt et al. obtained cryo-EM reconstructions of a ribosome stalled on the SARS-CoV-2 pseudoknot, showing how the pseudoknot lodges at the mRNA entrance channel and exerts tension to promote frameshifting; mutations that reduce pseudoknot stability reduce this effect, consistent with altered frameshifting efficiency.^26^ Other studies in SARS-CoV-2 and HIV contexts have used compensatory mutagenesis or stabilizing/destabilizing mutants to show that even modest destabilization in loops or base pairs in pseudoknot stems, for example, reduces frameshift frequency.^23,27^ These results clarify that both local structural stability and global pseudoknot topology critically modulate −1 PRF efficiency.

Given the conservation of PRF among alphaviruses, including CHIKV, Semliki Forest virus, and Sindbis virus, targeting this mechanism may have broad-spectrum antiviral potential.^28^ Accounting for kinetics by computational simulations offers a complementary view by stochastically modeling helix nucleation and kinetic traps as the transcript emerges to reveal competing early forming stem loops or pseudoknots.^29^ Our goal here is to develop a computational pipeline for predicting folding products and learn how to manipulate them to balance kinetic and thermodynamic factors.

A similar integrative approach was previously applied by our lab to dissect the regulatory landscape of the SARS-CoV-2 FSE. Using equilibrium and kinetic modeling frameworks, Schlick et al. first demonstrated that the viral RNA adopts a dynamic ensemble of stem loop and pseudoknot structures that shift depending on flanking sequences and cellular context.^15,30^ Building on this, Lee et al. showed that conformational switches in the SARS-CoV-2 FSE are modulated not only by the downstream pseudoknot but also by upstream RNA elements, revealing a cascade-like regulatory mechanism that coordinates ribosomal frameshifting with transcript elongation.^16^ Yan and Schlick recently suggested how spacer region tension triggers the conformational transition from one pseudoknot to another in the FSE.^18^ These studies led to the computational engineering of structure altering mutants.^31^ Because experiments confirmed the nearly abolished frameshifting of these mutants, such strategies appear useful.^27^ Here, we extend these ideas and insights to the CHIKV FSE to uncover conserved principles and virus-specific distinctions in RNA-mediated frameshifting control. Importantly, we probe cotranslational folding and test and iterate upon mutant design by this approach to achieve successful computational design. Subsequent experiments are ongoing to test this approach.

The outline of our paper is as follows. First, to establish a reliable method, we benchmark RNA prediction tools against known viral FSEs. Although no RNA 2D structure predictor tool is perfect, we identify NUPACK as a robust choice for our analysis. Second, we map the FSE’s equilibrium conformational landscape, revealing which structures are most thermodynamically stable as the flanking sequence is extended. This analysis identifies five core structural motifs, which we classify using our RNA-As-Graphs (RAG) framework into distinct topological families (e.g., the 3_6 pseudoknot vs the 2_2 stem loop; see Figure 2 and Materials And Methods). Third, guided by these equilibrium insights, we design four sets of targeted mutations intended to force the FSE into specific conformations. Fourth, to test if these designs are viable in a more realistic cellular context, we perform cotranslational folding simulations with KineFold. These kinetic simulations reveal whether a designed structure will form correctly as the RNA is synthesized or become trapped in an off-target state, providing a crucial validation of our design strategy. We reiterate on the design as needed to achieve the desired outcome. Our combined computational pipeline—thermodynamic conformational landscape prediction followed by analysis of the cotranslational ribosome/FSE, folding and refolding kinetics, mutant design, and subsequent computational testing and redesign—establishes a generalizable kinetics-informed RNA engineering platform.

Throughout this work, we use dual graph labels *V*_*n*_ such as 3_6 and 2_2 to refer to motif names in our RNA-As-Graphs approach, where *V* is the number of vertices and *n* is a motif index. See Figure 2 and Materials and methods for details. These coarse-grained motif definitions are needed to formulate our conformational landscapes and to design mutants using our dual graph machinery. For easy reference, we also provide arc plots for these topologies.

## Materials and Methods

### Computational Tools and Parameters

Many 2D structure prediction tools for RNA are available, and all are imperfect. Our goal by using them is to identify a robust tool that agrees with experimental data when available and makes reasonable predictions. No choice is perfect, and other tests are needed to further assess all predictors. All RNA secondary structure predictions and simulations were performed using the following software packages and parameters.

- NUPACK (v3.2.2):^33^ This package calculates minimum free energy (MFE) structures and equilibrium partition functions using a thermodynamic model based on dynamic programming. For landscape analysis, we used the subopt command with the -pseudo flag to generate an ensemble of suboptimal structures within a 3% energy gap of the MFE. Note that NUPACK does not support general pseudoknots (i.e., in its default ensemble it excludes crossing base-pairs), so there is a known limitation. However, for the CHIKV FSE systems we study, relatively simple or nested pseudoknots are involved, so NUPACK predictions are broadly consistent with those from pseudoknot-capable tools in our internal tests (see Supporting Information). We note, however, that no single method can serve as a standard.
- KineFold:^29^ This algorithm simulates cotranslational folding kinetics using a stochastic Monte Carlo approach to model helix formation as the RNA sequence emerges. All simulations were run using the kinefold_long_static executable with a fixed random seed of 12345, a transcription rate of 10 ms/nt, and a total simulation time of 3000 μs.
- RNAstructure (v6.2):^34^ This package is a versatile suite of tools used for a wide range of RNA secondary structure prediction and analysis tasks. In this work, it was primarily used for initial structural analysis and for generating visualizations of secondary structures.
- ipknot (v1.0.1):^35^ This program predicts pseudoknotted structures by formulating the folding problem as an integer programming task, decomposing complex topologies. In our usage, we invoked ipknot with default settings: level = 2 (i.e., nested pseudoknots only), scoring model = McCaskill, and no refinement (fast mode) (per the IPknot help documentation). These defaults restrict the pseudoknot class to nested forms, which is appropriate for the FSE pseudoknots we examine; in preliminary testing, alternative parameter settings (e.g., level = 3) produced qualitatively similar motif topologies. We include this detail here for full reproducibility.

The following programs were used exclusively for the comparative benchmarking analysis described in Section 3.1 and were run with their default parameters.

- ShapeKnots:^36^ This program extends standard thermodynamic folding algorithms by incorporating SHAPE reactivity data as pseudoenergy constraints to improve prediction accuracy.
- ProbKnot:^37^ This tool identifies the most probable secondary structure, including pseudoknots, by using base-pairing probabilities derived from a partition function calculation.
- Knotty:^38^ This program uses an exact minimum free energy pseudoknot secondary structure predictor, sparsified for efficiency.
- PknotsRG:^39^ This program predicts RNA secondary structures with a more restricted pseudoknot class using a dynamic programming approach, allowing near-optimal solutions and efficient analysis of long sequences through sliding windows.
- vsfold5:^40^ This tool uses a heuristic algorithm based on identifying the most stable stems to predict complex, multiloop pseudoknots.
- pKiss:^41^ This algorithm is specifically designed to predict kissing-loop interactions (a type of pseudoknot) by combining thermodynamic stability with a graph-theory-based approach.
- Vfold2D:^42,43^ This web server predicts RNA secondary structures, including pseudoknots, using a coarse-grained model and a loop-entropy-based energy function.

### Dual Graph Notation and Background

Our RNA-As-Graphs approach uses coarse-grained graphs to represent 2D RNA folds that share common motif features. Although our 2D folding predictions are performed in full sequence space, the graph motifs are used here (1) to describe conformational landscapes compactly, and (2) to define and design target topologies. In the dual graph representation, each continuous stack of base pairs (stem) is mapped to a vertex, and each intervening unpaired region (stem loops, bulges, internal loops, or multibranch junctions) is mapped to an edge connecting the two stems it separates.^44^ The advantage of RAG is that the same topologies can be associated with different base pair interactions as long as the stem loop motifs are the same. Thus, many 2D patterns can map onto the same RAG topology, and this coarse graining is advantageous for motif partioning, enumeration, design, and analysis. See reviews such as Schlick (2018)^45^ on the RNA-as-Graphs framework, Vicens & Kieft (2022)^46^ on conceptual models of RNA structure, and Schlick & Yan (2023)^47^ on combining structural and evolutionary perspectives.

Formally, for an RNA secondary structure with stems *S*_1_, ..., *S*_*k*_, ..., *S*_*n*_ and loops *L*_1_, ..., *L*_*m*_, where *k* refers to a stem index, the dual graph is *G* = (*V*, *E*) with

1 $$V=\left\{v_{i}:\;S_{i}\;is\;a\;stem\right\}$$

2 $$E=\left\{e_{ij}:\;L_{j}\;connects\;stems\;S_{i}\;\text{and}\;S_{k}\right\}.$$

An edge *e*_*ij*_ therefore exists whenever loop *L*_*j*_ directly bridges two stems, say *S*_*i*_ and *S*_*k*_; multiple edges or cycles in *G* correspond to pseudoknotted interactions.

In addition to mapping secondary structures to dual graphs, we also apply graph partitioning to decompose complex topologies into smaller, functionally relevant subgraphs (Figures 6b and 7b).^48^ For instance, large motifs such as 6_119 can be parsed into constituent elements—e.g., a 4_20 scaffold plus an embedded 2_2L FSE without breaking junctions or pseudoknots—revealing how modular folds assemble from simpler units.

### Sequences and Conformational Landscapes

The genome sequence of CHIKV was retrieved from GenBank (ID: NC_004162.2). Initial structural analysis and visualization were supported by the RNAstructure suite.^34^ All predicted secondary structures from our pipeline were subsequently converted into dual-graph representations for topological classification using our in-house RNA-As-Graphs (RAG) framework. The base 43 nt sequence (7 nt slippery site +36 nt FSE) was extended one nucleotide at a time, from 1 to 100 nt, either upstream or downstream, and the sequences were all analyzed using NUPACK (Figure 3).^33^

**Figure 3:**
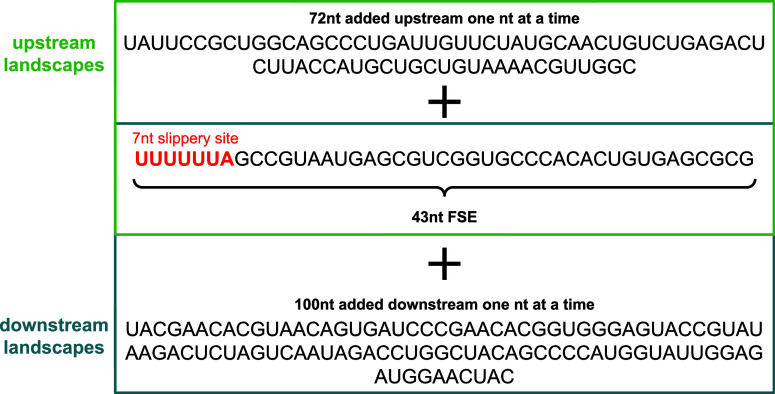
Workflow for generating landscapes.

For each CHIKV FSE extension, we used NUPACK v3.2.2 in subopt mode with the -pseudo flag to generate RNA secondary-structure ensembles. Sequences were analyzed for lengths from 43 to 143 nt in downstream analysis, and from 43 to 115 nt in upstream analysis. The asymmetric upstream/downstream window is done to balance computational tractability with biological system relevance. NUPACK’s pseudoknot-capable algorithms scale steeply in memory and runtime (O­(N^4^)), which places a practical upper limit on the sequence length that can be analyzed without excessive timeouts. In our benchmarking, upstream extensions longer than ∼70 nt routinely exceeded feasible runtimes on our computing environment, while downstream extensions remained tractable up to ∼100 nt. This is likely because there are fewer pseudoknots in the downstream. region Importantly, this upstream window is still biologically meaningful: the ∼70 nt range comfortably covers all experimentally observed upstream regulatory stems associated with CHIKV and other alphavirus FSEs.

The NUPACK output contains each 2D structure’s predicted free energy *E*_*i*_. We then applied the Boltzmann distribution at 37 °C to compute the partition function *Z* and the probability *p*_*i*_ of each structure

3 $$Z=\sum_{i=1}^{N}e^{-E_{i}/\left(k_{B}T\right)},,p_{i}=\frac{e^{-E_{i}/\left(k_{B}T\right)}}{Z}$$

where *k*_B_ is the Boltzmann constant and *T* the absolute temperature.

To analyze the landscape, each predicted 2D structure was first converted to its dual graph representation using our RAG framework. The probabilities of all structures mapping to the same graph topology were then summed.

It is important to note that the dual graphs are only used to annotate the landscapes, but the landscapes are created in nucleotide- and base-pair-space. For visualization, we retained only motifs whose total probability was ≥1% in at least one construct length. We then identified the minimal (i.e., irreducible) FSE-containing motif within each of these representative structures and kept only those minimal motifs that reached a probability of ≥5%. These probability thresholds were chosen to filter out conformational noise and focus the analysis on the most thermodynamically significant and structurally relevant motifs, consistent with prior landscape analyses.

### Inverse Folding by RAG-IF

We employed our inverse-folding protocol, RAG-IF, to mutate the FSE sequence, guiding it to fold into targeted dual-graph motifs. The RAG-IF protocol involves three primary steps:^30^

1. Identification of Mutation Regions and Target Structures: First, we determine specific regions for mutation and define their desired 2D structural motifs.
2. Generation of Candidate Sequences via Genetic Algorithm (GA): Second, candidate sequences are produced through mutations using a genetic algorithm. GAs simulate natural evolutionary processes, involving repeated cycles of random mutation, crossover (recombination), and selection. During these cycles, only sequences demonstrating high fitness, measured by the lowest Hamming distance to the target, are retained.
3. Optimization of Mutated Sequences: Third, the pool of mutated sequences is optimized by sorting and selectively retaining only those sequences with minimal or essential mutations required to fold correctly into the target dual-graph motifs.

To efficiently screen the large number of candidates generated by the GA, sequences are initially folded using the fast pseudoknot prediction software ipknot,^35^ and promising candidates are then more rigorously evaluated with NUPACK. Sequences successfully folding into the intended structure undergo additional refinement, where nonessential mutations are systematically removed to achieve minimal mutation profiles.

To promote formation of the 3_6 pseudoknot FSE structure, we specifically target mutations at the 3′-strand residues of stems S1 and S2. These mutations disrupt the existing G-C base pairs, thereby destabilizing and inhibiting the competing formation of the 2_2 FSE structure.

To target the 2_2L left-shifted stem loop FSE, we introduced precise substitutions on the 3′-strand residues of the primary stem, effectively reregistering its pairing interface by one nucleotide. These changes forge new Watson–Crick contacts that stabilize the shifted stem loop (2_2L) and simultaneously abolish the key base pairs required for the 3_6 FSE, thereby locking the element into the 2_2L topology.

To design the 2_2R right-shifted stem loop FSE, we introduced mutations on the 5′-strand of the core nested stem to shift its register two nucleotides downstream. By breaking the original G–C base pairs that nucleate the long-range pseudoknot and establishing new complementary contacts for the shifted stem, these changes abolish 3_6 folding and lock the element into the 2_2R topology.

Finally, to form the 2_1 FSE, we disrupted the native base pairs in both the proximal and distal stems (formerly defining the 2_2 fold) by mutating their 3′-strand residues and introduced complementary changes that realign these regions into a single contiguous helix. This strategy abolishes the competing 2_2 and 3_6 structures and locks the FSE into the 2_1 arrangement.

### KineFold Cotranslational Simulations

To model the kinetic aspects of FSE folding, we use KineFold, a well-established tool for simulating cotranslational folding pathways, noted for its ability to handle formation of complex topologies like pseudoknots, central to FSE function.^29^ Its stochastic simulation approach allows for the exploration of kinetic traps and competing folding pathways that cannot be captured by equilibrium thermodynamic models alone. The utility of KineFold and similar kinetic simulators for dissecting complex RNA folding landscapes has been highlighted in several studies and reviews, making it a suitable choice for testing the kinetic viability of our designed mutants.^49,50^

KineFold stochastically samples secondary-structure formation as the RNA is synthesized.^29^ For CHIKV, we ran kinefold_long_static with a fixed random-number seed of 12345 to ensure reproducibility. Transcription was modeled at a rate of 10 ms per nucleotide (100 nt/s). This rate was chosen as it falls within the experimentally measured range of 10–100 nt/s for viral and cellular RNA polymerases, providing a realistic time scale for observing cotranslational folding kinetics. The total simulation time was set to 3000 μs. With a transcription time of 1430 μs for the 143 nt sequence (43 nt FSE + 100 nt downstream), this provided a 1570 μs post-transcriptional window. This buffer allows for the observation of brief but meaningful structural rearrangements that may occur after transcription ceases but before downstream events like ribosome engagement. Pseudoknots were enabled, and all other KineFold parameters were left at their default settings.

KineFold tracks the instantaneous free energy (kcal/mol) of the minimal-energy structure at each sampling interval. The algorithm dynamically predicts the occupancy of all possible helices as they form and dissolve during the simulation. For our analysis and visualization, we selected the five most persistent or structurally significant helices identified by KineFold to serve as reporters for key folding events.

## Results

To orient the reader, we first present our integrated computational workflow (Figure 4), which links thermodynamic landscape prediction, inverse-folding design, and cotranslational kinetic validation. In the following subsections, we begin with computational benchmarking of 2D predictors, then move to conformational landscape mapping, mutation design, and kinetic simulation.

**Figure 4:**
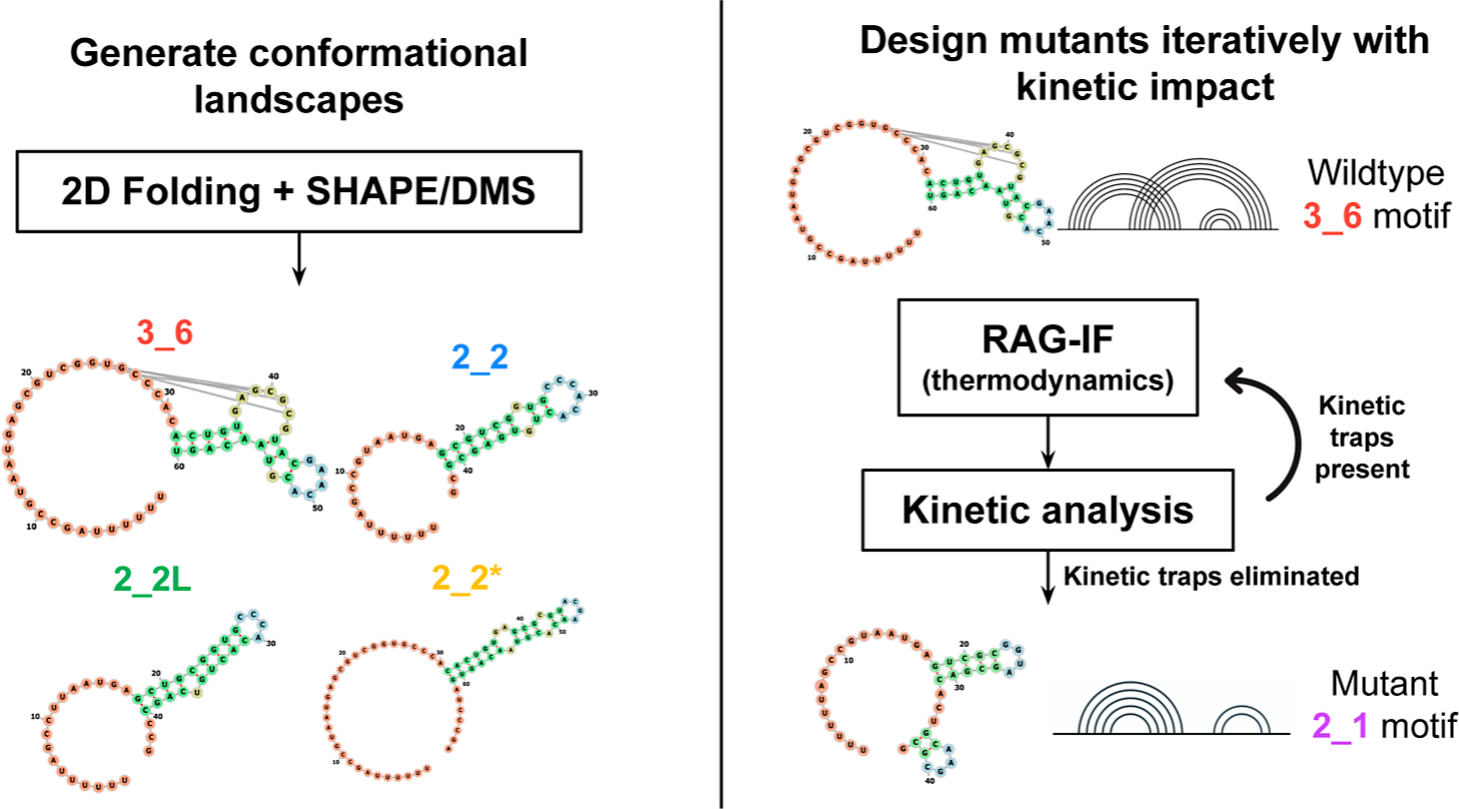
Integrated computational pipeline for kinetic-informed RNA design. This schematic outlines the iterative workflow combining thermodynamic landscape prediction, inverse folding mutation design (RAG-IF), and kinetic validation (KineFold) to engineer conformationally controlled RNA mutants. Step 1 identifies dominant folds in the wildtype CHIKV frameshift element (FSE) landscape; Step 2 applies RAG-IF to design mutations that shift folding toward a desired motif; and Step 3 reiterates design after kinetic validation to refine mutants whose folding trajectories deviate from the target conformation. Mutations with favorable folding kinetics and stability are retained as computationally validated candidates.

### Computational Benchmarking of 2D Predictors

Secondary structure prediction of FSE RNAs is critical for understanding frameshifting mechanisms; yet different algorithms often produce different results. To identify the most reliable predictor for our CHIKV analysis, we systematically benchmarked a total of nine programs across two viral contexts, first testing all nine against the experimentally validated SARS-CoV-2 FSE and then comparing a subset of five on the CHIKV FSE. Clearly, no program is perfect, but we sought a robust and reliable package that works for simple pseudoknots.

### CHIKV FSE

The five RNA 2D structure prediction tools we use that can predict pseudoknots are NUPACK,^33^ ShapeKnots,^36^ IPknot,^35^ ProbKnot,^37^ and Knotty^38^ (Figure 5). Each of the five RNA structure prediction tools here employs a distinct strategy for modeling pseudoknotted structures. See Materials and Methods for more details on how each program works.

**Figure 5:**
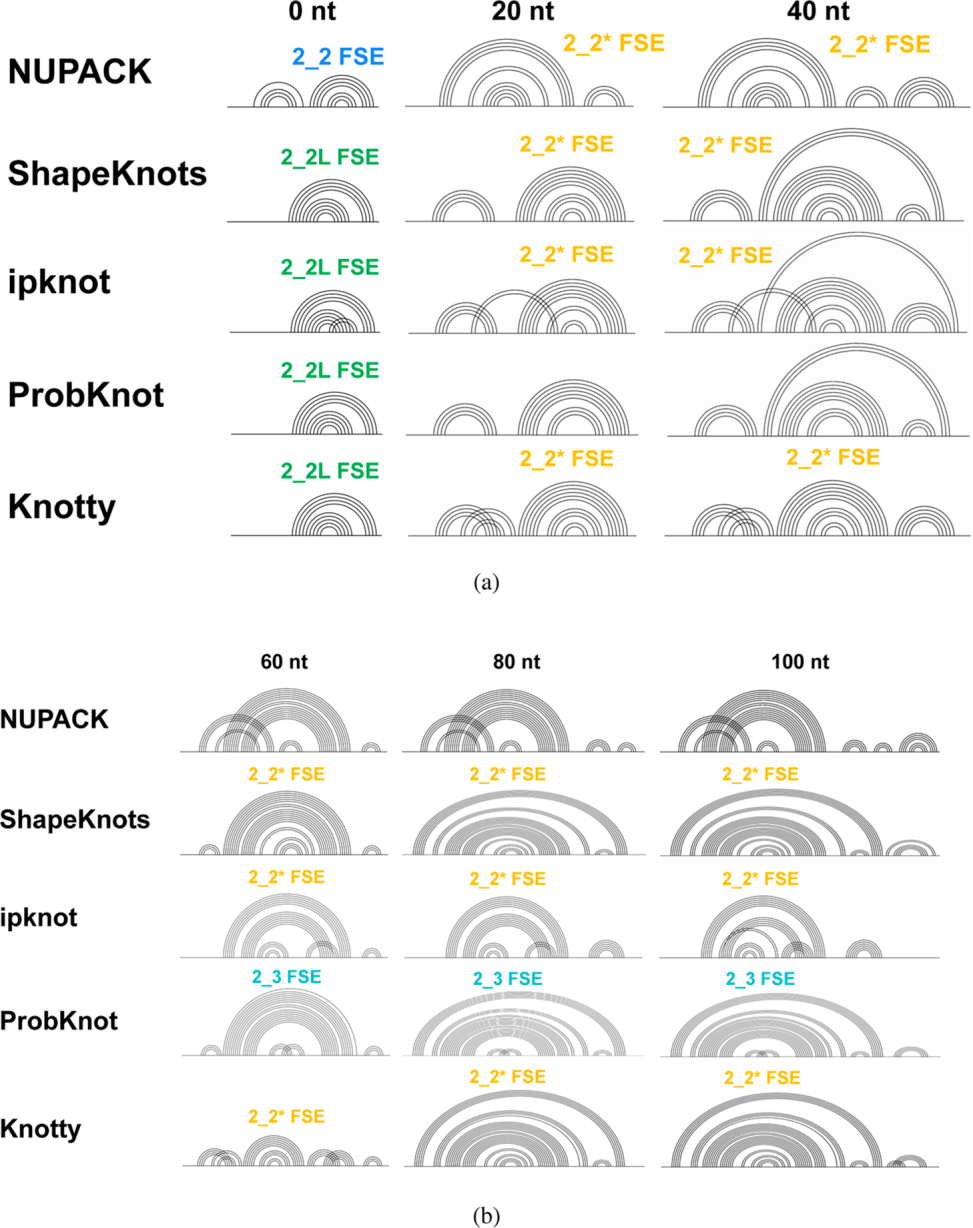
Predicted secondary structures for the CHIKV FSE for five computational methods (NUPACK, ShapeKnots, ipknot, ProbKnot, and Knotty) at increasing sequence length. (a) Minimum free energy (MFE) structures at 0, 20, and 40 downstream nucleotides. (b) MFE structures at 60, 80, and 100 downstream nucleotides. Each structure is annotated with its corresponding FSE motif (e.g., 2_2 FSE, 2_2L FSE).

For each tool, we extracted four key structural metrics that characterize FSE functionality at six upstream extension lengths of the CHIKV FSE (0, 20, 40, 60, 80, and 100 nt):

1. BP = number of base pairs (base-pairing density)
2. Stems = number of contiguous runs of paired bases (structural organization)
3. AvgStem = average stem length (BP/Stems, structural complexity)
4. Pk = pseudoknot presence (topological complexity)

To quantify how dramatically these structural predictions diverge as sequence context increases, we calculated the Hamming distance between every pair of program predictions at each extension length. The Hamming distance measures the number of differing positions between two dot-bracket structures, providing a direct metric of prediction disagreement. Formally, for two strings *x* = *x*_1_ *x*_2_...*x*_*n*_ and *y* = *y*_1_ *y*_2_...*y*_*n*_

4 $$d_{H}\left(x,y\right)=\sum_{i=1}^{n}1\left(x_{i}\neq y_{i}\right)$$

where 1(·) is 1 if its argument is true and 0 otherwise. We use the raw Hamming distance to quantify the absolute increase in structural disagreement as sequence context expands. Added flanking nucleotides introduce new positions where folding predictions can diverge, and raw distances directly capture this effect. Length normalization would mask this expansion and compress the trend of divergence.

Table 2a summarizes the structural metrics, while Table 2b reports the mean and maximum Hamming distances across all ten program pairs.

**Table 2:** Base-Pair Metrics for CHIKV FSE Predictions at Selected Downstream Extensions for (a) Five 2D Structure Programs and (b) Comparative Analysis among Them

(a) quantitative base-pair analysis of CHIKV FSE predictions across selected 3′ extensions.						
program	nt	BP	stems	AvgStem	Pk	Motif
NUPACK	0	11	4	2.75	false	2_2
	20	14	4	3.50	false	2_2*
	40	19	5	3.80	false	2_2*
	60	27	6	4.50	true	Other
	80	31	7	4.43	true	Other
	100	38	9	4.22	true	Other
ShapeKnots	0	9	1	9.00	false	2_2L
	20	15	4	3.75	false	2_2*
	40	21	6	3.50	false	2_2*
	60	28	7	4.00	false	2_2*
	80	34	8	4.25	false	2_2*
	100	40	10	4.00	false	2_2*
ipknot	0	11	2	5.50	true	2_2L
	20	16	5	3.20	true	2_2*
	40	23	7	3.29	true	2_2*
	60	24	6	4.00	true	2_2*
	80	27	6	4.50	true	2_2*
	100	29	7	4.14	true	2_2*
ProbKnot	0	9	1	9.00	false	2_2L
	20	13	3	4.33	false	Other
	40	19	5	3.80	false	Other
	60	28	6	4.67	true	2_3
	80	35	7	5.00	true	2_3
	100	39	8	4.88	true	2_3
Knotty	0	9	1	9.00	false	2_2L
	20	19	6	3.17	true	2_2*
	40	24	7	3.43	true	2_2*
	60	31	9	3.44	true	2_2*
	80	34	8	4.25	false	2_2*
	100	41	10	4.10	true	2_2*

(b) structural divergence across five 2D structure predictors (hamming distance)		
nt	mean	max
0	8.8	19
20	18.7	39
40	25.1	44
60	48.0	71
80	50.9	80
100	72.3	98

The results reveal striking patterns in both individual tool behavior and interprogram agreement. NUPACK shows predictable scaling behavior: base-pairing increases steadily from 11 to 38 pairs, stem count rises monotonically, and pseudoknots appear only when substantial upstream context is provided (≥60 nt). ipknot predicts pseudoknots even at minimal extensions (0–20 nt) and shows more erratic scaling patterns.

As expected, the Hamming distance analysis reveals that prediction disagreement grows exponentially with sequence length. At 0 nt extension, programs differ by an average of only 9 positions, but by 100 nt, the average disagreement exceeds 72 positions—nearly half the total sequence length. Maximal pairwise distances reach 98 positions, indicating that some program pairs produce almost entirely different structural predictions for the same sequence.

To ground these computational predictions in experimental data, we compared the MFE structures from each tool to the SHAPE-MaP-informed model of the FSE proposed by Madden et al.^6^ Interestingly, none of the purely computational MFE predictions perfectly recapitulated the experimentally derived structure, which features a specific stem loop conformation. This discrepancy highlights the inherent challenge of predicting RNA structure from sequence alone and underscores the value of integrating experimental constraints. It also motivates our approach of analyzing the entire conformational ensemble rather than relying on a single MFE prediction.

Figure 5 visualizes the striking divergence in predictions across the five programs as sequence length increases. At the core FSE length (0 nt), most tools predict a simple stem loop, but ipknot already identifies a pseudoknot. As the sequence is extended, the predictions diverge significantly. ShapeKnots consistently predicts simple, pseudoknot-free structures, while ipknot, ProbKnot, and Knotty predict increasingly complex pseudoknotted topologies, even with minimal added context (20–40 nt). In contrast, NUPACK demonstrates a more conservative and context-dependent behavior: it predicts simple stem loops at shorter lengths and only introduces the more complex 3_6 pseudoknot when sufficient downstream sequence is available (at 60 nt and beyond). This predictable scaling, where structural complexity increases with sequence length, aligns well with the expected behavior of cotranslational folding and makes NUPACK a suitable choice for generating the CHIKV FSE conformational landscapes in this study.

### SARS-CoV-2 FSE

We further assess NUPACK against eight other secondary-structure prediction tools to determine which programs reliably recover the experimentally validated 3_6 pseudoknot fold of the wildtype SARS-CoV-2 FSE (Table 3). For each 2D structure program that predicts pseudoknots, we extracted the minimum-free-energy (MFE) structure and converted it into dual-graph notation for direct comparison against the crystal-derived 3_6 pseudoknot motif.^16^

**Table 3:**
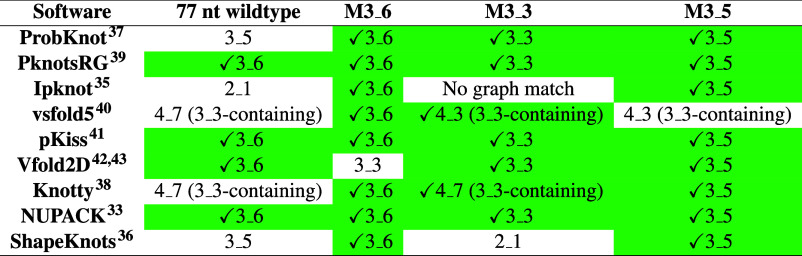
Secondary Structures Predicted by Eight Software Packages for SARS-CoV-2 77 nt FSE and Motif Strengthening Mutants Confirmed Experimentally^a^ ^,^ ^27,30^

a MFE structures are presented in dual graph notations.

Prediction accuracy is scored as “correct” only when the dual-graph of the MFE fold exactly matches the known 3_6 topology. As summarized in Lee et al.^16^ and Table 3, this benchmark confirms that PknotsRG, pKiss, NUPACK, and Vfold2D consistently recover the canonical structure for the wildtype FSE. Table 3 also tests prediction accuracy of the programs for three structure stabilizing mutants confirmed experimentally.^27,30^ This highlights the effectiveness of PknotsRG, pkiss, and NUPACK. Together, with performance on the CHIKV FSE (Section 3.2), we consider NUPACK’s favorable scaling and its conservative approach—avoiding the prediction of complex pseudoknots without sufficient stabilizing sequence context—appropriate. These tests suggest that NUPACK is robust and reliable for generating the CHIKV conformational landscapes in the remainder of this study.

### Downstream Conformational Landscapes

We generate conformational landscapes of frameshifting element-containing RNA sequences computationally to analyze the various conformations of the CHIKV systems as a function of increasing sequence length, mimicking ribosomal translation. Thus, adding upstream (5′) residues mimics the RNA sequence that the ribosome encounters when the genome is unwound, as the ribosome moves along the RNA transcript. For comparison, we also constructed landscapes with varying downstream (3′) sequence lengths to mimic the refolding when the ribosome moves further downstream.

We begin by analyzing the conformational landscape of CHIKV systems as a function of increasing sequence length, one downstream nt at a time (Figure 6a).

**Figure 6:**
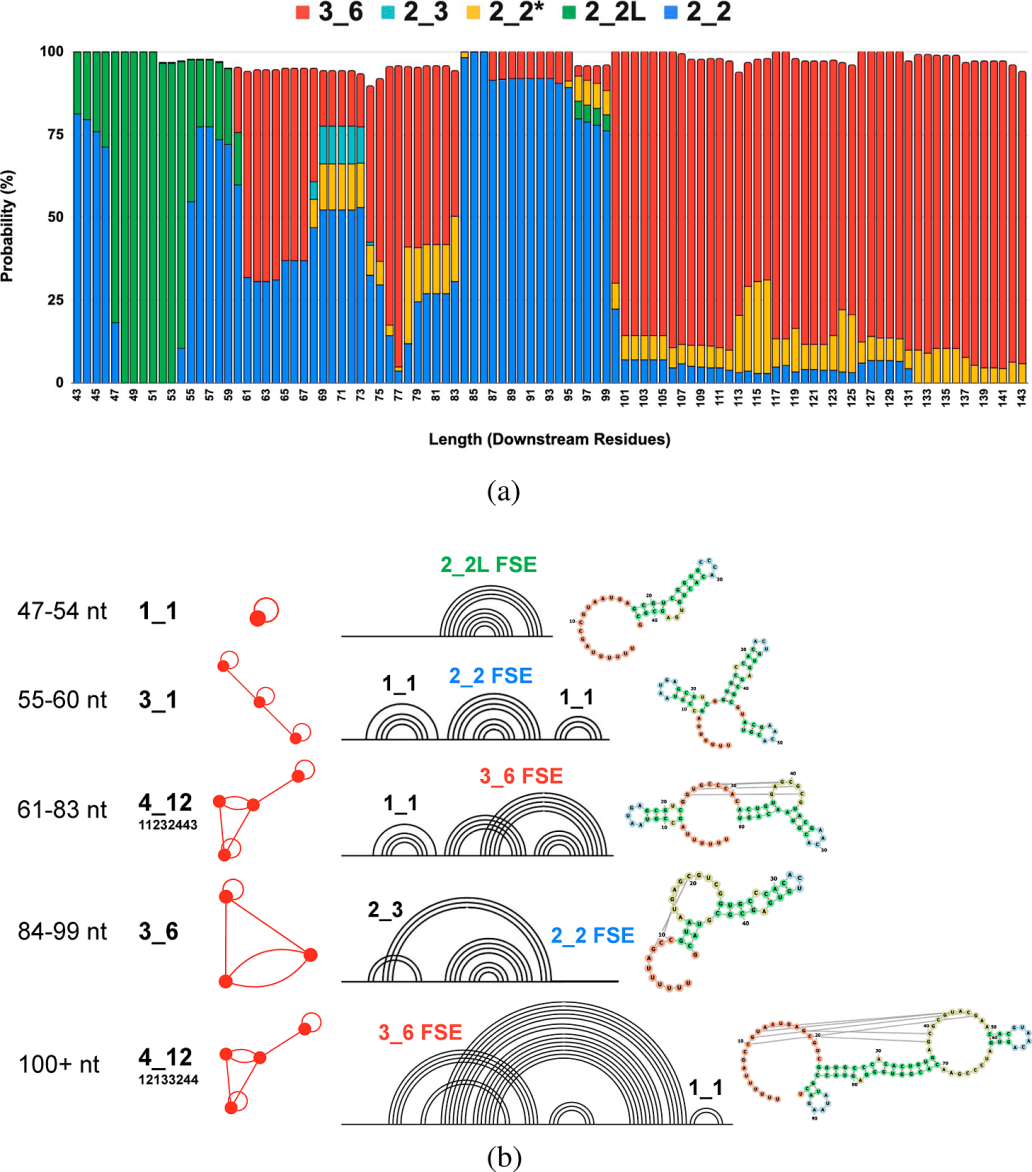
Structural and topological features of the CHIKV FSE. (a) Distribution of conformations highlighting heterogeneous regions. (b) Dominant structural topologies across different downstream lengths and their respective subgraphs.

At short lengths (43–54 nt), 2_2 motifs initially dominate, but 2_2L motifs take over at 47 nt. Here, the most dominant motif is a simple stem loop: 1_1 (Figure 6b), which contains the 2_2L FSE. At 55 nt, the 3_1 motif becomes the dominant structure, but it is not that distinct from 1_1, as it is only 3 stem loops, and a 2_2 FSE instead of a 2_2L.

At 61 nt, the FSE forms a 3_6 pseudoknot plus another stem (1_1 + 3_6), forming a 4_12 motif. The transition from motif 3_1 to motif 4_12 is driven by the formation of a new pseudoknot-like interaction, introduced by the additional AACAGU nucleotides at the 3′ end. In 3_1 at 55–60 nt, the 2D fold is primarily composed of a single stem loop and a nested stem loop, whereas in 4_12, the new nucleotides establish long-range base-pairing with an upstream region, stabilizing the overall conformation. This motif maintains dominance for the remainder of the landscape. Significantly, the emergence of this 3_6 pseudoknot as the thermodynamically favored structure aligns perfectly with experimental studies that have identified a conserved, functional pseudoknot as being essential for CHIKV frameshifting.^8,10^

### Upstream Conformational Landscapes

We now analyze the conformational landscape of CHIKV systems as a function of increasing sequence length, one upstream nt at a time as a measure of RNA refolding as the ribosome moves away (Figure 7a).

**Figure 7:**
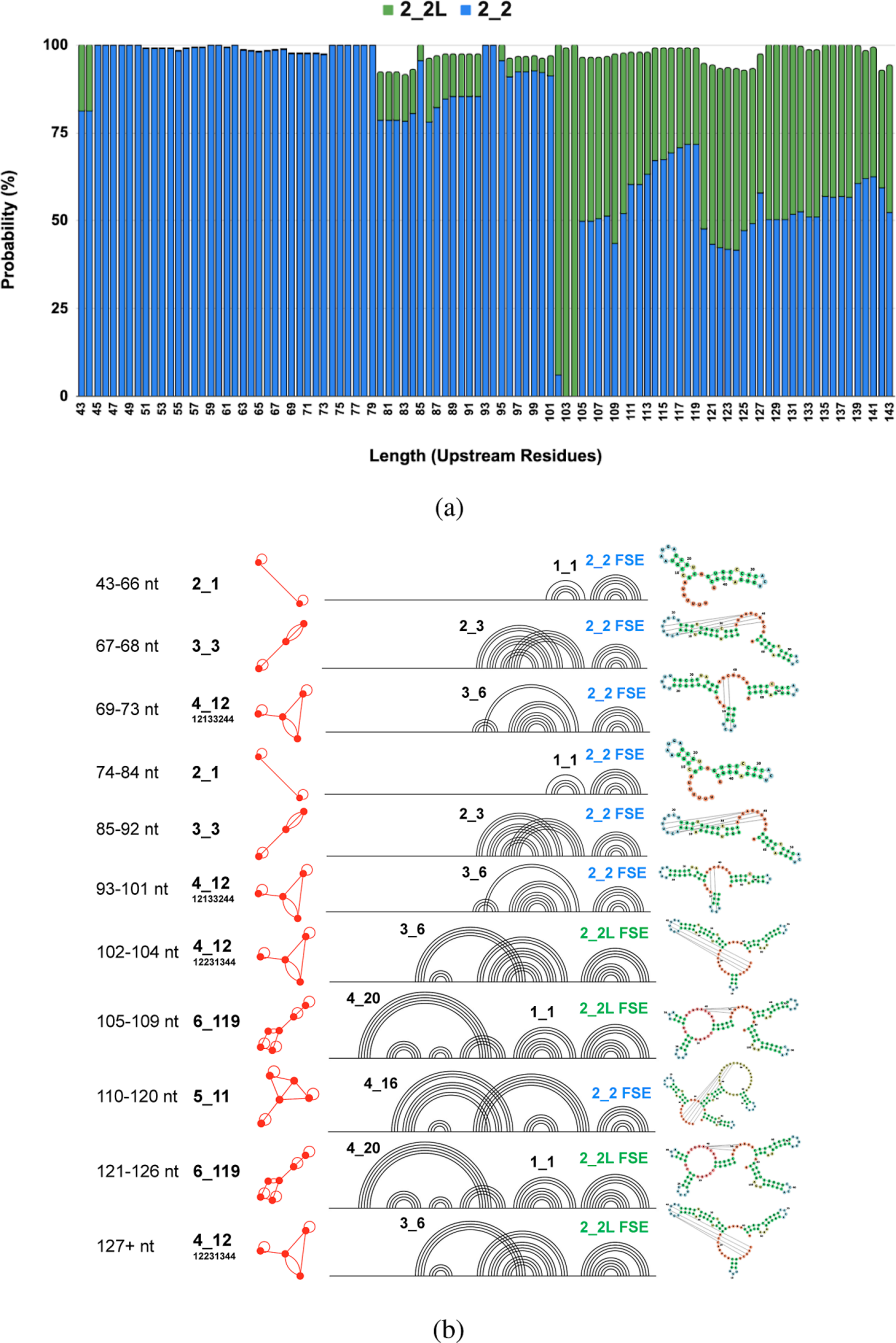
Structural and topological features of the CHIKV FSE with upstream sequence extensions. (a) Conformational diversity highlighting dominance of 2_2 motifs. (b) Topological evolution as a function of upstream sequence length.

As the CHIKV FSE sequence lengthens, distinct topological structures progressively emerge, each characterized by increasingly complex base-pairing interactions (Figure 7b). Initially, from 43–66 nt, the structure forms a simple 2_1 topology. With sequence expansion (67–68 nt), a slightly more complex 3_3 pseudoknot topology arises. Further extensions (69–73 nt) yield the more intricate 4_12 topology, defined by nested interactions including the characteristic 3_6 pseudoknot plus a simple stem loop. As the sequence lengthens further (74–104 nt), structural shifts alternate between simpler topologies (2_1, 3_3) and the stable 4_12 topology (93–104 nt), demonstrating its persistence. Beyond 104 nucleotides, even more complex topologies, such as 6_119 and 5_11, emerge (105–120 nt), characterized by extensive base-pairing patterns and long-range interactions, producing pseudoknots along with other stems. Ultimately, at lengths of 121 nt and beyond, the topology stabilizes into the 6_119 form (see decomposition of complex motifs in Figure 7b).

The emergence of the 2_2L motif in the 102+ nt range marks a significant shift from the previously dominant 2_2 motifs observed throughout the CHIKV FSE structural landscape. This transition may occur as upstream nucleotides near the 2_2 FSE establish new interactions with recently added residues, creating the structural flexibility needed for the 2_2L FSE to emerge. However, at 110+ nt, the 2_2 FSE regains dominance due to the increasing structural constraints imposed by additional upstream residues, which may outcompete the stabilizing interactions that favor the 2_2L conformation; yet the 2_2L FSE does not disappear completely.

### Mutation Design

Programmed ribosomal frameshifting (PRF) in alphaviruses such as CHIKV is tightly regulated by structured RNA elements, including pseudoknots and stem loops near the slippery sequence. Alterations to these structures can markedly influence frameshifting efficiency, thereby affecting expression of downstream viral proteins such as the transframe (TF) protein. Prior studies have demonstrated that mutations within the FSE can either enhance or suppress PRF. Similarly, Kendra et al. used SHAPE-MaP-guided mutagenesis to show that disruptions to a stem loop in the 6K region impacted ribosomal pausing and reduced frameshifting efficiency.^8^ These findings underscore the potential of targeted mutations to reprogram FSE conformational dynamics and shift the structural equilibrium toward or away from frameshift-favorable conformations. In this section, we use our RAG-IF protocol to design four mutants intended to bias the FSE toward specific target conformations: pseudoknot 3_6 (red), nested stem loop 2_2L (green), nested stem loop 2_2R (orange), or two separated stems 2_1 (purple) (see motifs in Figures 2, 8, and 9; see Materials and Methods for more detail on how each mutant was designed; see Supporting Information Section S1 for mutant sequences). Our validation of these designs is a two-step process. First, we use NUPACK equilibrium analysis to confirm that the mutations successfully reshape the thermodynamic landscape to favor the intended structures; see Figures 8 and 9. Second, we perform cotranslational simulations in the following Section 3.5 to test the kinetic accessibility of these target folds; see results in Figure 10.

**Figure 8:**
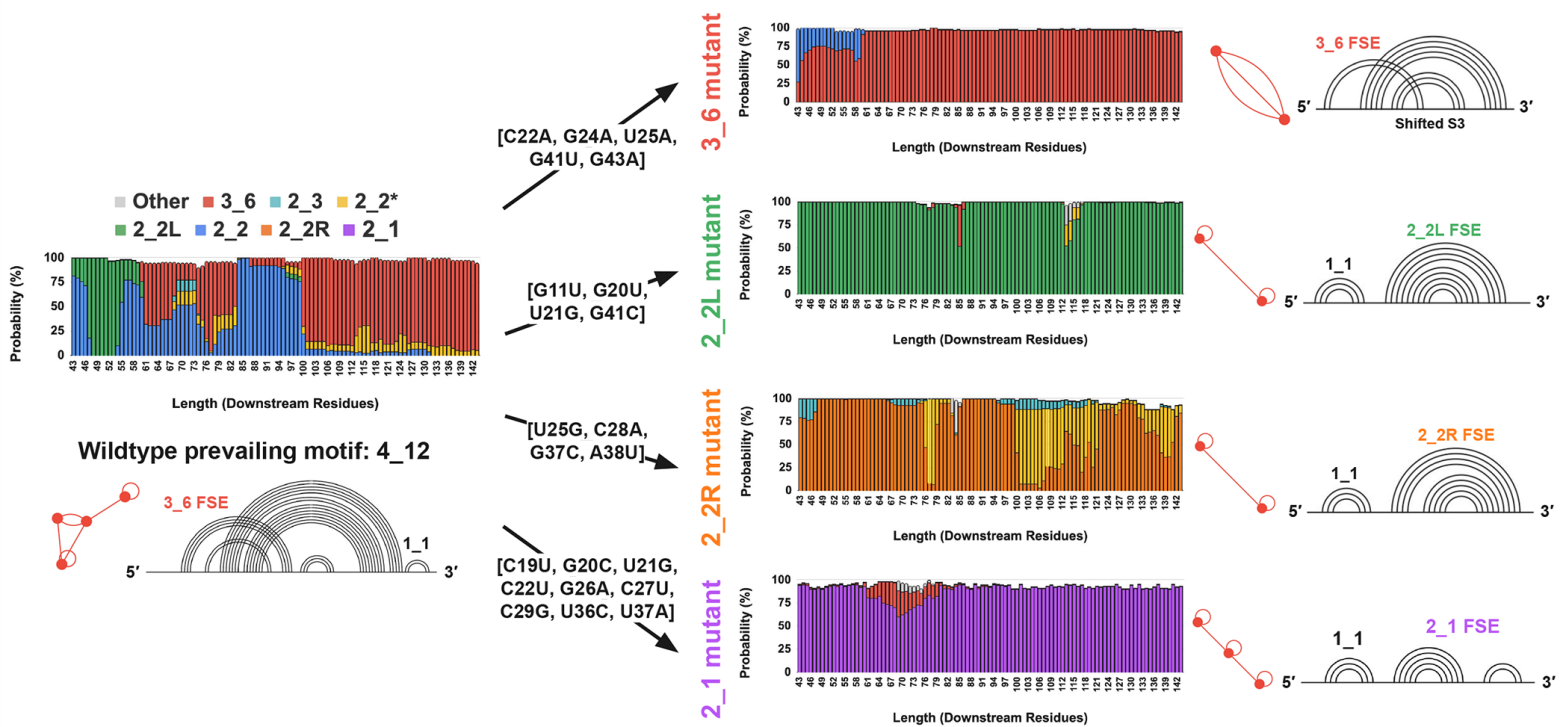
Downstream folding landscapes of wildtype and mutant CHIKV FSEs. Conformational probabilities are plotted as a function of downstream sequence length (43–142 nt) for the wildtype CHIKV FSE (left) and four designed mutants (right). Wildtype folding converges on the 4_12 motif containing a 3_6 FSE (red), whereas each mutant biases the ensemble toward an alternative target conformation: 3_6 (red), 2_2L (green), 2_2R (orange), or 2_1 (purple). For each mutant, the prevailing graph motif and its component substructures are annotated alongside the representative dual graph and arc diagram. Mutation sets are listed adjacent to each construct.

**Figure 9:**
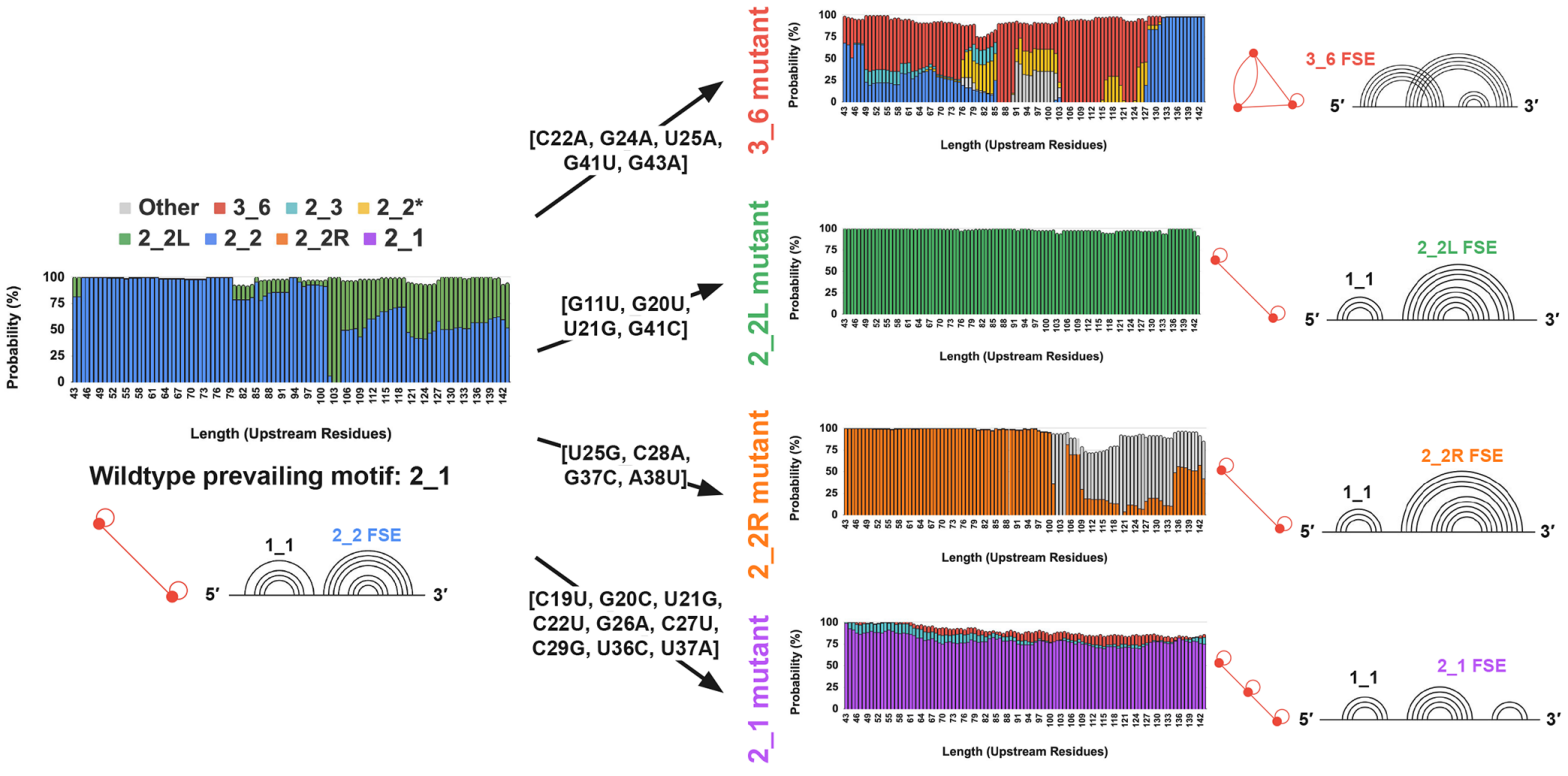
Upstream folding landscapes of wildtype and mutant CHIKV FSEs. Conformational motif probabilities are shown as a function of upstream sequence length (43–142 nt) for the wildtype CHIKV FSE (left) and four engineered mutants (right). The wildtype predominantly adopts the 2_1 topology with a 2_2 FSE, while mutations redirect folding toward distinct targets: 3_6 (red), 2_2L (green), 2_2R (orange), and 2_1 (purple). Mutation sets are listed adjacent to each construct.

**Figure 10:**
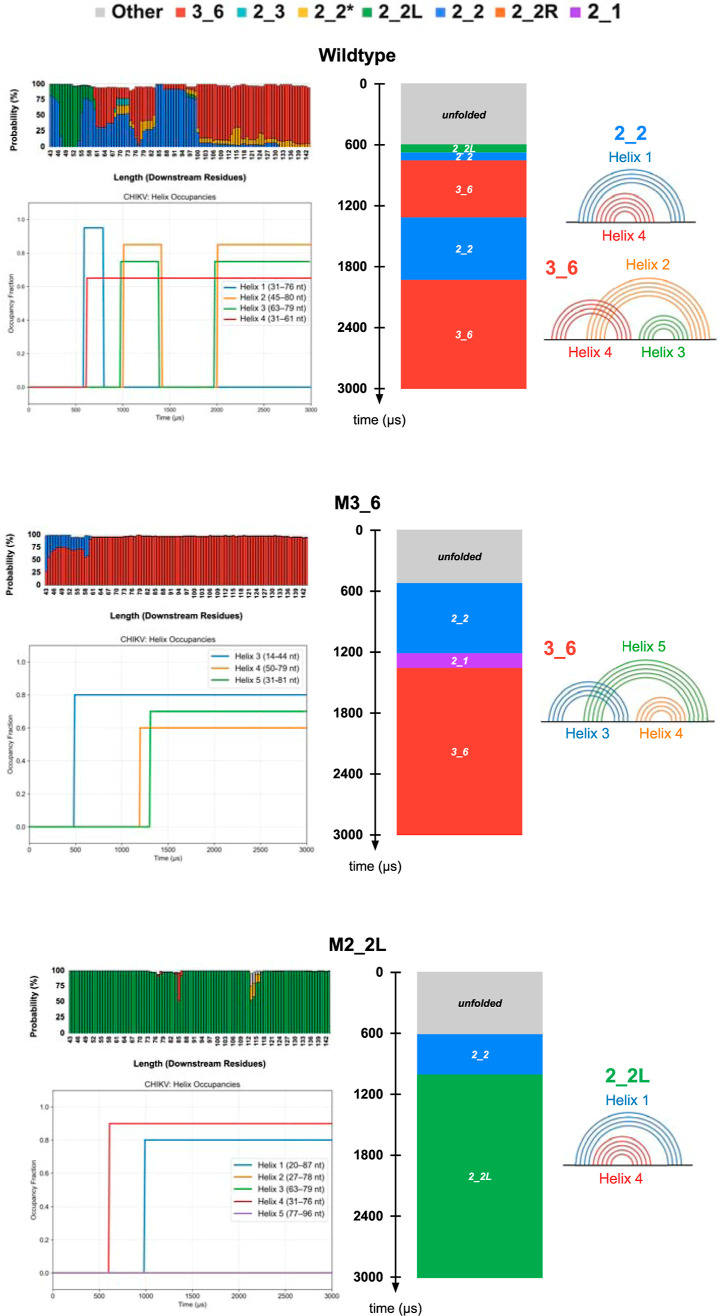

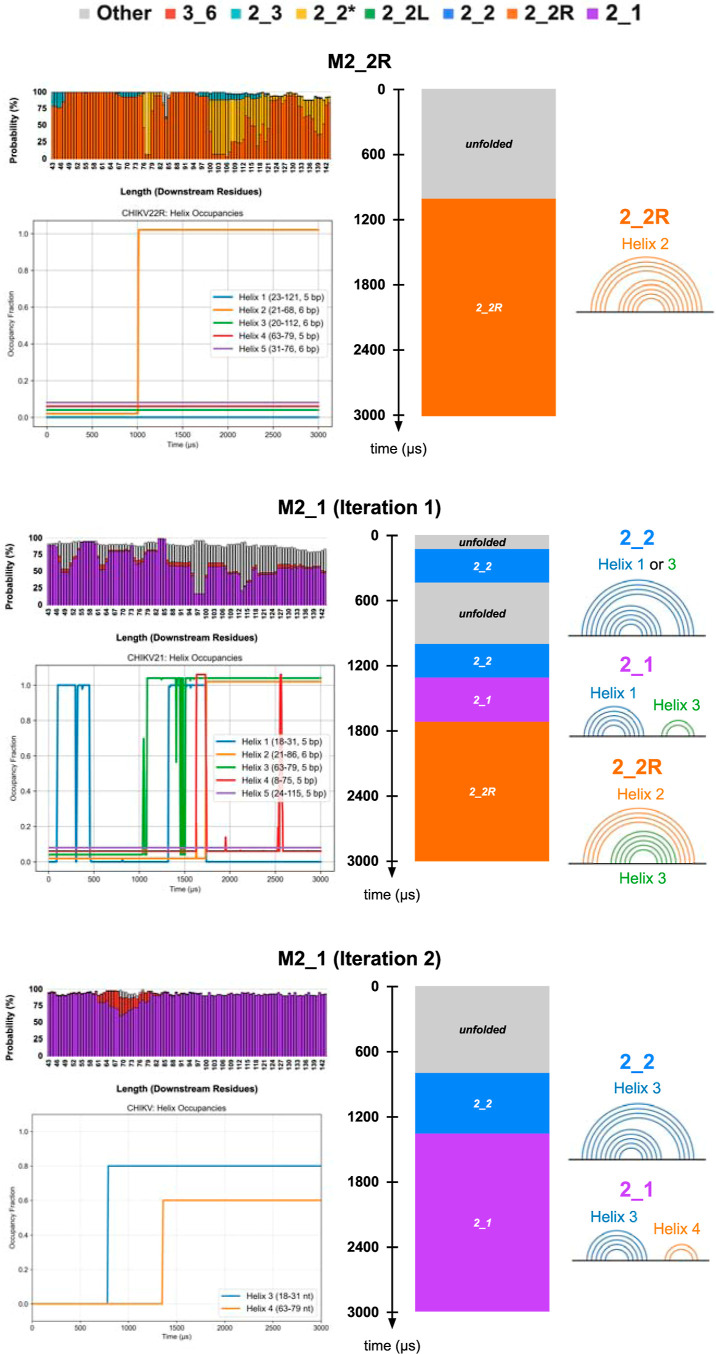
Co-translational folding trajectories of CHIKV FSE variants with structural-motif key. (Top) Small color legend illustrating the structural motifs used throughout the analysis. (Rows) KineFold simulation results for (first row) the wildtype construct and the 3_6 mutant; (second row) the 2_2L and 2_2R mutants; and (third row) the original 2_1 construct (see more in SI section S3) alongside its redesigned 2_1 variant. Each panel comprises: (1) equilibrium conformational probabilities across downstream sequence lengths; (2) helix occupancy fractions for tracked stems; and (3) a coarse-grained summary of dominant structural motifs over time.

#### 3_6 FSE Mutation Design (M3_6)

Motivated by this regulatory plasticity, we introduce a five-nucleotide mutation into the CHIKV FSE specifically designed to favor formation of the 3_6 pseudoknot, a structure we hypothesize to be more conducive to efficient −1 PRF compared to the competing 2_2 stem loop and its longer variant, 2_2L. This strategy aims to experimentally bias the FSE folding landscape and test the functional consequences of pseudoknot stabilization on translational recoding efficiency.

When applied to downstream sequences, this mutation reshapes the folding energy landscape, consistently favoring the 3_6 FSE across all sequence lengths, particularly for sequences shorter than 100 nucleotides. However, the 2_2 FSE remains slightly preferred at shorter lengths (under approximately 60 nucleotides). The overall folding probability for the 3_6 FSE increases significantly from 50.43% in the wildtype sequence to 94.53% in the mutant sequence.

For upstream sequences, the same five-nucleotide mutation promotes formation of the 3_6 pseudoknot structure instead of the competing 2_2 and 2_2L FSEs. While this mutation does not completely reshape the folding energy landscape as dramatically as it did for downstream residues, the 3_6 pseudoknot is still favored at virtually all sequence lengths. The overall folding probability for the 3_6 FSE increases significantly from 0.00% in the wildtype sequence to 51.59% in the mutant sequence.

#### 2_2L FSE Mutation Design (M2_2L)

We introduce a four-nucleotide mutation [G11U, G20U, U21G, G41C] into the CHIKV FSE designed specifically to strongly promote formation of the 2_2L stem loop structure instead of the competing 3_6, 2_3, 2_2*, and 2_2 FSEs.

When applied to downstream sequences, this mutation dramatically reshapes the folding energy landscape, consistently favoring the 2_2L FSE across virtually all sequence lengths. The overall folding probability for the 2_2L FSE increases significantly from 10.34% in the wildtype sequence to 98.16% in the mutant sequence, representing a nearly complete shift in folding preference.

For upstream sequences, the same four-nucleotide mutation exhibits even more pronounced effects, completely eliminating competing structures and promoting exclusive formation of the 2_2L pseudoknot structure. The overall folding probability for the 2_2L FSE increases from 21.96% in the wildtype sequence to 100.00% in the mutant sequence, achieving complete structural dominance across all sequence lengths.

#### 2_2R FSE Mutation Design (M2_2R)

Our quadruple nucleotide substitution [U25G, C28A, G37C, A38U] within the CHIKV FSE biases the folding pathway toward the 2_2R stem loop. This design is intended to dominate over alternative conformations such as 3_6, 2_3, 2_2, 2_2L, and the canonical 2_2 pseudoknots.

In the wildtype CHIKV FSE folding landscape, the 2_2R motif is never populated under physiological conditions, meaning that introducing it can effectively lock the RNA into a conformation that is hypothesized to be nonproductive for frameshifting, making it an interesting candidate for therapeutic development. Structurally, the 2_2R stem loop differs from the 2_2L form in that its internal stem loop is displaced toward the 3′ end of the sequence rather than toward the 5′ end, thereby altering the register of stem loop interactions and preventing the left-shifted folding seen in the 2_2L construct.

Applied to downstream sequence contexts, these four mutations substantially remodel the folding energy landscape and drive almost exclusive adoption of the 2_2R motif. In the wildtype, the 2_2R structure is never observed (0.00% probability), whereas in the mutant, its prevalence rises to 75.21% across nearly all length variants—a pronounced reorientation of the folding equilibrium.

When evaluated within upstream sequence fragments, the same nucleotide changes likewise enforce the 2_2R fold by abolishing competing folds. Here, the probability of the 2_2R motif jumps from 0.00% in the native sequence to 72.68% in the engineered construct.

#### 2_1 FSE Mutation Design (M2_1)

To enforce the 2_1 two-stem conformation—a topology never sampled in the native energy landscape—we engineered a nine-nucleotide substitution set: [C19U, G20C, U21G, C22U, G26A, C27U, C29G, U36C, U37A]. This set was designed to simultaneously disrupt the base pairing of all competing native folds (3_6, 2_2, etc.) and to create a stable interaction pattern required for the 2_1 topology.

The designed mutations proved highly effective at reconfiguring the thermodynamic landscape. In the downstream context, where the wildtype FSE never populates the 2_1 motif (0.00% probability), the M2_1 mutant exhibits near-total dominance of this target structure, reaching an integrated probability of 95.24%. This reflects a complete reorientation of the folding equilibrium toward the engineered conformation.

A similarly dramatic effect was observed in the upstream sequence contexts. There, the native sequence again shows no 2_1 population (0.00%), whereas the engineered construct drives the 2_1 motif to a dominant 87.66% overall probability. In both downstream and upstream landscapes, all competing native structures are almost eliminated, collapsing to negligible probabilities.

This result highlights the utility of the RAG-IF design pipeline. By introducing a set of coordinated changes, it is possible to not only suppress native, competing structures but also to drive the folding equilibrium almost completely toward a desired, non-native conformation, highlighting the potential for rational, structure-based design of RNA elements.

### Predicted Effects on Frameshifting Efficiency

To synthesize these structural findings into functional expectations, we summarize the predicted effects of each engineered mutant on frameshifting efficiency in Table 4, based on structural dominance and prior empirical studies of PRF-related conformations.

**Table 4:** Predicted Effects of CHIKV FSE Mutations on Frameshifting Efficiency^a^

mutant	dominant structure	predicted effect on PRF	justification
M3_6	3_6 pseudoknot	↑ increase	matches known PRF-promoting motif^10,11^
M2_2L	2_2L nested stem loop	↔ slight reduction	maintains motif but may alter dynamics^13^
M2_2R	2_2R nested stem loop	↓ decrease	novel conformation, likely nonproductive^12,32^
M2_1	2_1 two-stem conformation	↓↓ strong decrease	never observed natively, likely disrupts PRF^?^

a Each mutant was designed to favor a specific RNA pseudoknot structure, thereby shifting the conformational ensemble of the FSE. Based on prior studies, the 3_6 pseudoknot is expected to promote efficient −1 PRF,^10,11^ while alternative structures such as 2_2L, 2_2R, and 2_1 are predicted to reduce or eliminate frameshifting.^12,13,32^

The 3_6 pseudoknot is associated with more stable and complex RNA tertiary structures, which are known to enhance ribosomal pausing and promote frameshifting. Hanson et al. identified the 3_6 pseudoknot as a key regulatory motif supporting efficient −1 PRF in CHIKV through SHAPE-MaP probing.^10^ Additionally, Diaz demonstrated that pseudoknots involving kissing-loop interactions like those found in 3_6 enhance frameshifting through mechanical tension on the ribosome.^11^ The large increase in folding probability (from ∼50% to >94% downstream) suggests a strong shift toward a high-efficiency PRF conformation. We engineer the 3_6 mutant to demonstrate that our design strategy can successfully stabilize a known frameshifting motif in silico, providing a baseline for evaluating other designs.

Although 2_2L is a variant of the canonical 2_2 stem loop, its extended stem and loop architecture may either support or slightly hinder ribosome stalling depending on its stability. The work of Zimmer et al. showed that subtle changes in loop register and extension can alter pseudoknot-induced pausing and efficiency.^13^ Since this conformation still contains elements of a pseudoknot, some frameshifting is likely preserved, but the redistribution away from 3_6 may reduce peak efficiency. The increase in probability to ∼98% suggests structural dominance, but whether this conformation is as effective mechanistically remains speculative.

The 2_2R stem loop is not naturally sampled in wildtype CHIKV, and its shifted internal stem loop register may fail to induce the mechanical resistance required for efficient frameshifting. Firth & Atkins highlighted that deviations from conserved PRF motifs significantly diminish efficiency in alphaviruses.^32^ Moreover, Tants & Schlundt identified specific structural signatures—such as bulged nucleotides and kinked stems—that are essential for optimal PRF,^12^ which may be disrupted in 2_2R. Because the 2_2R fold was previously unobserved (0.00%) and is now enforced, it may act as a nonproductive, decoy conformation, likely resulting in frameshifting suppression.

The 2_1 two-stem conformation is also not detected in the wildtype ensemble and requires eight coordinated mutations to become accessible. As with 2_2R, the lack of prior natural occurrence suggests it may disrupt ribosome pausing or induce premature translation. Kendra et al. showed that targeted point mutations in the CHIKV −1 PRF signal can dramatically reduce frameshifting efficiency in cell-based reporter assays.^8^ Therefore, this mutant is predicted to significantly disrupt or abolish efficient frameshifting, consistent with its design to suppress competing folds and enforce a previously inaccessible structure.

### Co-translational Folding Kinetics

While the equilibrium landscapes in the preceding sections confirm that our designed mutants are thermodynamically biased toward their target structures, this analysis does not account for the process of synthesis itself. In a cell, an RNA molecule folds cotranslationally, meaning local structures whose sequences emerge early can form rapidly. These structures can become long-lived “kinetic traps,” potentially preventing the formation of the globally optimal structure if it requires long-range interactions that are not yet available. This competition between fast, local folding and slower, global rearrangement is a fundamental principle of RNA biology that can cause the functional, in vivo structure to differ from the one with the lowest predicted free energy.

To address this critical question of kinetic accessibility and test the robustness of our designs, we performed cotranslational folding simulations for the wildtype and each mutant using KineFold.^29^ This analysis allows us to directly compare the predicted thermodynamic end point with the more realistic cotranslational folding pathway.

KineFold simulates cotranslational RNA folding by modeling the stochastic formation of secondary structures during sequential nucleotide addition, mimicking RNA polymerase elongation one base at a time. It uses a Gillespie-like algorithm to sample kinetic folding pathways based on experimentally calibrated base-pairing transition rates and free energy changes. At each elongation step, KineFold evaluates accessible structural rearrangements, allowing it to capture not only the final equilibrium conformation but also transient intermediates, misfolded states, and kinetic traps that may emerge during synthesis. This provides a mechanistic view of how structure formation is influenced by transcription order and folding kinetics.^29^

For each construct, we defined five candidate helices based on their recurrence across ensemble predictions and tracked their nucleation and persistence over simulated transcription time (for more helix information and free energy discussions, see Supporting Information section S2 and Figure S1). This approach allows us to observe when specific stems form, whether they coexist or displace one another, and how early forming structures may trap the transcript in metastable states. In the following sections, we present KineFold trajectories for five constructs—wildtype, 3_6 mutant, 2_2L mutant, 2_2R mutant, and 2_1 mutant—linking free-energy descent and stem occupancy to specific folding intermediates (Figure 10). These kinetic profiles are then compared against equilibrium predictions to evaluate how transcriptional context shapes the conformational ensemble.

### Wildtype FSE

To characterize the cotranslational folding trajectory of the wildtype CHIKV frameshifting element (FSE), we simulated RNA elongation from 43 to 143 nucleotides using KineFold. Five candidate helices were tracked throughout the run:

- Helix 1: bases 31–76 (7 bp)
- Helix 2: bases 27–78 (5 bp)
- Helix 3: bases 63–79 (5 bp)
- Helix 4: bases 31–61 (6 bp)
- Helix 5: bases 21–131 (8 bp)

Helix 1 (blue) is the first to form, briefly rising to full occupancy at *t* ≈ 500 μs before disappearing entirely after *t* ≈ 800 μs. Around *t* ≈ 900 μs, Helices 2 (orange), 3 (green), and 4 (red) enter the ensemble together, with Helix 2 reaching occupancy near 0.9, Helix 3 near 0.75, and Helix 4 near 0.65. Helices 2 and 3 are lost at *t* ≈ 1500 μs but reform again beginning around *t* ≈ 1900 μs and remain dominant through the end of the trajectory. This pattern indicates a metastable triad of coexisting helices that represent the core of the wildtype FSE’s kinetically accessible structural ensemble.

Taken together, these data define a hierarchical, partially reversible folding cascade for the wildtype CHIKV FSE under cotranslational conditions:

1. Early (0–500 μs; length < 30 nt): no stable helices; the RNA remains largely unstructured.
2. Helix 1 nucleation (500–800 μs; length ≈ 31–76 nt): Helix 1 rapidly forms and reaches full occupancy, then disappears entirely after *t* ≈ 800 μs.
3. Multistem formation (900–1500 μs; length ≈ 27–79 nt): Helices 2, 3, and 4 form together and persist with high occupancy, driving the free energy down to ∼−0.22 kcal/mol.
4. Transient rearrangement (1500–1900 μs): Helices 2 and 3 are briefly lost, while Helix 4 remains stable, resulting in a temporary plateau in energy descent.
5. Restabilization (1900–3000 μs): Helices 2 and 3 reform and coexist with Helix 4, further lowering the free energy to ∼−0.28 kcal/mol.

This kinetic profile reveals a structured folding pathway in which Helix 4 acts as a persistent scaffold, while Helices 2 and 3 exhibit dynamic entry and exit from the ensemble. The absence of Helix 5 throughout suggests that long-range stems are kinetically disfavored, and that CHIKV’s FSE primarily explores modular, short-range stem loop topologies during transcription.

These kinetic insights dovetail directly with the static, downstream-length landscapes shown in Figure 6a,b. As the transcript reaches ∼50–55 nt, the kinetic trajectory transitions out of an unfolded ensemble and begins to sample 2_2 and 2_2L stem loop motifs, which are also prominent in equilibrium predictions at these lengths. Between *t* ≈ 600–1200 μs, the system adopts the 3_6 pseudoknot, reflecting early pseudoknot accessibility as soon as Helix 3 becomes transcribable. Interestingly, this is followed by a pronounced return to the 2_2 motif between *t* ≈ 1200–1800 μs, suggesting that the initial pseudoknot state is transient and that stem-looplike folds can outcompete it kinetically, even after pseudoknot nucleation. In the final third of the trajectory (*t* > 1800 μs), the system re-enters and stabilizes the 3_6 state, which then persists through the remainder of the simulation. This late-stage pseudoknot adoption mirrors the dominant 3_6 motif predicted in equilibrium at full transcript length, but the intervening competition with 2_2 illustrates that cotranslational folding does not follow a single downhill energy pathway—instead, it toggles between metastable conformations before settling. This observation provides a compelling mechanistic explanation for the varied structures reported in the literature (Table 1); experimental approaches may be capturing either the transiently formed kinetic traps (e.g., stem loops) or the final thermodynamic ground state (e.g., pseudoknots), depending on the specific constructs and conditions used.

Together, these results demonstrate that the CHIKV FSE folding pathway is not simply a reflection of the minimum free energy structure at each length, but a kinetic progression shaped by transcriptional timing and stem accessibility. Short-range stem loops such as 2_2 may transiently outcompete pseudoknots like 3_6, delaying their full adoption until later time points. This underscores the role of kinetic gating in modulating conformational outcomes and suggests that in vivo, CHIKV’s frameshift efficiency may be strongly influenced by the rate of transcription and the order in which structural elements emerge.

### M3_6 Mutant

To evaluate how targeted nucleotide substitutions influence cotranslational folding of the CHIKV FSE, we analyzed a five-nucleotide mutant [C22A, G24A, U25A, G41U, G43A] designed to stabilize the 3_6 pseudoknot motif (see Section 3.6.1). KineFold simulations were run using the same transcription window (43–143 nt).

Helix occupancies confirm the effectiveness of this mutant. Helix 3 (blue) forms earliest, stabilizing at occupancy ∼0.9 before *t* = 500 μs and persisting throughout the trajectory. Helices 4 (orange) and 5 (green) both nucleate around *t* = 1200 μs and remain stably occupied at ∼0.6–0.7, consistent with persistent pseudoknot formation. All three helices remain co-occupied through the end of the simulation, indicating successful structural stabilization by design.

Taken together, these observations define the following folding cascade:

1. Early (0–500 μs): RNA is unfolded; no helices have formed.
2. 2_2 intermediate (500–1200 μs): A transient stem loop forms, corresponding to 2_2 topology in the coarse-grained classification.
3. Helix 4 formation (1200–1400 μs): Another stem loop stabilizes, briefly producing a 2_1 dual-graph motif.
4. Helix 5 formation and pseudoknot consolidation (1400–3000 μs): The 3_6 motif dominates the structure through the remainder of the trajectory.

Comparison with the equilibrium NUPACK ensemble reveals partial divergence at early transcript lengths. While NUPACK predicts strong 3_6 pseudoknot dominance across nearly all downstream lengths, KineFold simulations show that the cotranslational pathway initially passes through 2_2 and 2_1 motifs—likely due to their shorter stems and earlier accessibility. However, by *t* ≈ 1400 μs, both approaches converge: the kinetic pathway locks into the 3_6 motif and maintains it through to the final transcript length, matching the equilibrium pseudoknot prediction. This transient disagreement followed by long-term agreement underscores how cotranslational folding intermediates may differ from static ensemble predictions, even when the final adopted structure aligns.

### M2_2L Mutant

To evaluate how rational design can enforce stem-loop-dominated folding trajectories, we analyzed a CHIKV mutant [G11U, G20U, U21G, G41C] designed to stabilize the 2_2L motif (see Section 3.6.2). Co-translational folding simulations were performed using KineFold over a 43–143 nt transcription window.

Helix occupancy data confirm this mutant’s efficacy as well. Helix 4 (red), which forms part of the early 2_2 intermediate, rises rapidly at *t* ≈ 600 μs and remains occupied throughout. Helix 1 (blue), which spans 20–87 nt and defines the long stem loop of the 2_2L motif, sharply increases in occupancy starting at *t* ≈ 1000 μs and maintains near-unity occupancy through the end of the simulation. The remaining helices (2, 3, and 5) show minimal or negligible sampling.

This trajectory defines the following folding cascade:

1. Early (0–600 μs): RNA remains largely unstructured.
2. Helix 4 nucleation (600–1000 μs): A short-range stem loop (31–76 nt) forms, producing a 2_2 motif.
3. Helix 1 stabilization (1000–3000 μs): The long-range Helix 1 structure locks in, transitioning the ensemble to the 2_2L motif.

Comparison with the equilibrium ensemble reveals partial disagreement during early folding. While the conformational landscape predicts a dominant 2_2L motif across all downstream lengths, KineFold simulations show a transient detour through a 2_2 intermediate in the first 1000 μs. This discrepancy likely arises from cotranslational stem competition: Helix 4 (31–76 nt) becomes accessible before the full length of Helix 1 is transcribed, allowing it to transiently dominate. Once the 3′ segment of Helix 1 emerges, it overtakes Helix 4 and establishes the intended 2_2L conformation. Thus, while both models ultimately converge on the same structural outcome, the kinetic pathway highlights a brief stem loop intermediate that static predictions miss.

### M2_2R Mutant

To bias the CHIKV FSE toward a right-shifted nested stem loop topology, we simulated cotranslational folding of the 2_2R-favoring mutant [U25G, C28A, G37C, A38U] (Section 3.6.3). KineFold was run from 43–143 nt.

The resulting folding trajectory is straightforward:

1. 0–1000 μs: The RNA remains unfolded.
2. 1000–3000 μs: Helix 2 forms and defines a persistent 2_2R motif.

Notably, the cotranslational trajectory matches the equilibrium landscape almost exactly: both KineFold and NUPACK show exclusive dominance of the 2_2R structure across all relevant transcript lengths. This mutant represents the cleanest case of design-convergent structure formation in our study.

### M2_1 Mutant

Our initial thermodynamic design for a 2_1 motif, while promising at equilibrium, proved to be kinetically frustrated, converging to an off-target 2_2R structure in cotranslational simulations (Figure 9, M2_1 (Iteration 1); see Supporting Information Section S3 and Figure S2)). This provided a critical insight: an effective design must not only favor the target structure thermodynamically but also destabilize competing kinetic traps. Guided by this, we developed a new nine-nucleotide mutant, M2_1 [C19U, G20C, U21G, C22U, G26A, C27U, C29G, U36C, U37A], specifically engineered to overcome the previously observed kinetic barrier (see Section 3.6.4).

The helix occupancy plot for the new M2_1 mutant in Figure 10 reveals an improved and more direct folding pathway. After an initial unfolded period, the simulation shows a brief, low-occupancy formation of a transient 2_2-like intermediate. However, unlike the original mutant, this state is quickly resolved. Around *t* ≈ 1200 μs, the helices corresponding to the target 2_1 structure (Helices 3 and 4) rapidly form and lock in at near-unity occupancy, remaining stable for the rest of the simulation. Critically, the helices that would form the off-target 2_2R trap show no significant occupancy.

These data define a highly efficient folding cascade:

1. Unfolded State (0–600 μs): The RNA remains largely unstructured as it emerges.
2. Transient Intermediate (600–1200 μs): A short-lived, simple stem loop (2_2 motif) is briefly sampled at low probability.
3. Convergent Folding (1200–3000 μs): The system rapidly and decisively folds into the target 2_1 conformation, which remains stably populated throughout the remainder of the simulation.

This kinetic trajectory is now in excellent agreement with our thermodynamic predictions. The redesigned M2_1 mutant not only shows overwhelming stability for the 2_1 fold at equilibrium (95.24% downstream, 87.66% upstream) but, as these kinetic simulations confirm, it can also access this state efficiently during cotranslational folding without getting diverted into significant kinetic traps.

This result serves as a proof-of-concept of our iterative design strategy. The kinetic analysis of the initial, failed M2_1 mutant allowed us to identify the specific off-target helices responsible for forming the problematic 2_2R kinetic trap (in this case, helices 2 and 4). By applying this knowledge to refine our RAG-IF protocol with new negative constraints to suppress these helices, we successfully generated the improved mutant. This demonstrates how kinetic analysis can be used to diagnose the failures of a purely thermodynamic design and guide the rational engineering of mutations to achieve a desired folding outcome in both equilibrium and kinetic landscapes.

## Discussion

Our integrated computational analysis of the CHIKV frameshifting element (FSE) reveals how subtle variations in RNA sequence context and translational kinetics drive significant structural transitions that regulate – 1 programmed ribosomal frameshifting. We demonstrate that both upstream and downstream extensions can shift the folding equilibrium from simple stem loops to more complex pseudoknot topologies, particularly the functionally relevant 3_6 motif. These findings underscore the structural plasticity of the FSE and its potential vulnerability to targeted disruption, as highlighted previously.^19,20^

Our work helps reconcile the various structural models for the CHIKV FSE reported in the literature (Table 1). These studies, using different construct lengths and experimental methods, have reported stem loops, pseudoknots, and other intermediate folds. Our findings suggest these are not mutually exclusive observations but rather snapshots of a dynamic folding process governed by a competition between folding kinetics and thermodynamic stability. By modeling this process, we can define the specific conditions under which one force dominates the other, providing a unifying framework for these seemingly disparate results. Recent comparative analyzes of coronavirus frameshift elements have similarly emphasized that PRF efficiency emerges from shifts in the structural ensemble rather than a single dominant fold, reinforcing the idea that multiple conformations coexist and contribute functionally.^51^

So, when can a kinetic trap override the thermodynamically preferred structure? Our results indicate this occurs when a stable, local structure (like a simple stem loop) can form rapidly from a newly synthesized portion of the RNA sequence. This early forming structure creates a metastable “kinetic trap” because the energy barrier to unfold it is significant. The thermodynamically optimal structure (e.g., a complex pseudoknot) may have a lower free energy overall, but it cannot form until distant parts of the sequence are transcribed, by which time the RNA may already be locked in the kinetic trap. As the FSE mRNA transcript is associated with multiple ribosomes, folding and refolding continuously occurs as certain residues become accessible or occluded. Our wildtype FSE landscapes demonstrate that early forming stem loops dominate the folding pathway, delaying or even preventing the formation of the more stable 3_6 pseudoknot. This understanding can be exploited for the design of specific mutants that help suppress some folds while favoring others.

By introducing targeted mutations informed by kinetic analysis, we effectively enhance formation of the desired fold. The M3_6 mutant, for example, was so strongly biased toward the pseudoknot that it formed immediately upon synthesis, bypassing the competing stem loop traps entirely. This demonstrates that the outcome is not predetermined but is a tunable balance between the speed of local folding and the stability of the final, global structure.

A key insight from this work is that rationally designed mutations—altering as few as five nucleotides—can dramatically reshape the conformational energy landscape, biasing the ensemble toward pseudoknots that are otherwise inaccessible in the wildtype sequence. While these mutations do not universally eliminate competing motifs, they significantly elevate the population of pseudoknotted structures, offering a proof-of-concept for antiviral strategies that target FSE conformational bias.

Co-translational folding simulations reveal that kinetic constraints can strongly shape the structural trajectory of the CHIKV FSE, often diverging from equilibrium predictions. In the wildtype construct, early forming stem loops dominate initially, delaying or preventing access to pseudoknotted states favored at longer transcript lengths. The 3_6 mutant rapidly adopts a stable pseudoknot motif, demonstrating strong agreement between kinetic and thermodynamic models. The 2_2L mutant shows an early detour through a transient 2_2 state before committing to the intended 2_2L fold, highlighting how folding delays can arise from the order of nucleotide availability. The 2_2R mutant achieves near-perfect enforcement of its target structure, with the 2_2R motif dominating from the moment it becomes accessible. In contrast, the 2_1 mutant fails to retain its designed conformation, passing only briefly through the 2_1 state before ultimately converging on the off-target 2_2R motif. These results illustrate that cotranslational folding can either reinforce or frustrate design intent, depending on how effectively the target motif outcompetes alternatives during stepwise RNA synthesis. Accounting for these kinetic effects is essential for accurately predicting RNA behavior and designing functional structural elements.

While our successful designs of the 3_6, 2_2L, and 2_2R mutants provide strong support for the utility of the design pipeline, the initial kinetic failure of the 2_1 mutant and its reiteration to achieve success in design underscores that equilibrium predictions alone are insufficient for rational RNA design. The kinetically driven convergence of the 2_1 mutant to an off-target structure highlights the indispensable role of kinetic analysis in predicting functional outcomes and avoiding unintended structural fates.

Our findings thus highlight a fundamental distinction between thermodynamic and kinetic control of RNA folding. While equilibrium predictions identify 3_6 as the dominant low-energy pseudoknot, kinetic simulations reveal that early forming stem loop motifs such as 2_2L and 2_1 often act as folding traps, diverting trajectories away from the global minimum. This divergence underscores the importance of cotranslational context: as the RNA emerges, helix nucleation order and sequence availability bias the ensemble toward metastable intermediates. Conformational selection in the FSE is therefore shaped not only by free energy, but by local dynamics and competition among folds, suggesting new routes for modulating frameshifting through kinetic control.

Methodologically, our benchmarking of five structure-prediction algorithms reinforces the value of cross-validating predictions with experimental data and suggests that ensemble modeling, incorporating multiple predictors and SHAPE constraints, will yield more reliable structural hypotheses. Indeed, our SHAPE-informed calculations reduced the incidence of spurious pseudoknots and aligned more closely with known biological features. This supports a hybrid modeling paradigm where experimental constraints refine algorithmic predictions, bridging the gap between in silico inference and in vivo relevance.

Several limitations should be acknowledged. Both NUPACK and KineFold rely on thermodynamic and kinetic parameters derived from simplified in vitro systems, which may not fully capture the influence of cellular factors such as RNA chaperones, ribosomal stalling, or molecular crowding. Co-translational simulations assume constant elongation rates and do not model RNA polymerase pausing or backtracking, which could influence folding windows. Furthermore, while KineFold is a robust tool for this analysis, our study relies on a single kinetic model; future work could benefit from comparative analyzes using other cotranslational folding simulators, such as Kinfold from the ViennaRNA package or coarse-grained models like oxRNA, to ensure the predicted folding pathways are not model-dependent.^52,53^ Moreover, our inverse folding strategy does not consider tertiary interactions or noncanonical base pairs, which may stabilize functional motifs in vivo; further atomic molecular dynamics simulations are needed to ensure the validity of associated 3D models. Although our use of SHAPE-informed constraints mitigates some of these concerns, experimental validation—through mutational scanning, ribosome profiling, and high-resolution structural techniques—is essential to confirm the biological relevance of predicted conformations.

Besides structure validation, studies assessing the impact of our designed mutants on PRF efficiency, viral replication, and infectivity in cellular and animal models will clarify their therapeutic potential. Ultimately, structural characterization of FSE interactions with the ribosome will be important. Meanwhile, the integration of emerging deep-learning-based folding models may offer more comprehensive sampling of RNA structural ensembles. Ultimately, these insights could inform the rational design of small molecules or antisense oligonucleotides that stabilize or destabilize key FSE motifs, offering a novel route to CHIKV inhibition.

Taken together, our findings deepen the mechanistic understanding of FSE-mediated frameshifting and provide a generalizable computational framework for targeting RNA structural dynamics in viral genomes—a promising direction for next-generation antiviral therapeutics.

## Conclusions

We have presented an integrated thermodynamic and kinetic computational pipeline for the folding of the Chikungunya virus frameshifting element. We show that the FSE conformation adopts several 2D folds governed by a dynamic competition between thermodynamic stability and cotranslational folding kinetics. By mapping the FSE’s equilibrium and kinetic landscapes, we demonstrate that while the functionally critical 3_6 pseudoknot is thermodynamically favored at longer sequence lengths, the wildtype FSE is often kinetically trapped in simpler, more rapidly formed stem loop structures during synthesis. This finding helps reconcile the varied structures previously reported in the literature. Furthermore, we show that the FSE’s folding pathway can be rationally targeted to design four distinct FSE folds. Computational examination of these mutants by both equilibrium and kinetic simulations via molecular dynamics simulations along with experimental characterization will help reveal the complex interplay inherent in FSE landscapes and functional implications.

## Supplementary Material



## Notes

Construct sequences (WT and mutants: M3_6, M2_2L, M2_2R, M2_1 iterations); KineFold helices and free-energy traces for wildtype and mutant FSEs; Conformational landscapes and kinetic data of M2_1 mutants; Figures S1–S2: cotranslational folding traces and thermodynamic landscape comparisons (PDF)

## Notes

All data and analysis scripts are available on GitHub.
