# Supplementary material for: Kinetic Traps in RNA Folding: Targeted Design of Frameshifting Element Mutants by Thermodynamic and Kinetic Analysis of the Chikungunya Virus
Source: J Phys Chem B. 2026 Jan 29;130(6):1810–31. doi: 10.1021/acs.jpcb.5c08223 (PMC12908123; doi:10.1021/acs.jpcb.5c08223)
Supplement: Supplementary file 1 [file jp5c08223_si_001.pdf]

My New Template (2020), S1–S5

ARTICLE

Supporting Information for: Kinetic Traps in RNA Folding: Targeted Design of Frameshifting Element Mutants by Thermodynamic and Kinetic Analysis of the Chikungunya Virus

Samuel Lee[1] and Tamar Schlick[2,3,4,5,*]

[1]University of Pennsylvania, Philadelphia, PA, 19104, United States of America
[2]Department of Chemistry, New York University, New York, New York 10003, United States
[3]Courant Institute of Mathematical Sciences, New York University, New York, New York 10012, United States
[4]NYU-ECNU Center for Computational Chemistry, NYU Shanghai, Shanghai 200062, PR China
[5]NYU Simons Center for Computational Physical Chemistry, New York University, New York, New York 10003, United States

Corresponding author: schlick@nyu.edu

S1. Construct sequences

The following sequences were used for the equilibrium landscape and co-transcriptional folding analyses. The core 43-nucleotide region (7-nt slippery site + 36-nt FSE) is shown. For landscape generation, this core sequence was extended by 100 nt upstream or downstream using the native CHIKV genome sequence (GenBank ID: NC_004162.2). For M2_1, Iteration 1 was unsuccessful due to kinetic traps (see Figure 9).

```
>Wildtype (WT)
UUUUUUAGCCGUAAUGAGCGUCGGUGCCCACACUGUGAGCGCG

>M3_6 Mutant ([C22A, G24A, U25A, G41U, G43A])
UUUUUUAGCCGUAAUGAGCGUAGAAGCCCACACUGUGAGCUCA

>M2_2L Mutant ([G11U, G20U, U21G, G41C])
UUUUUUAGCCUUAAUGAGCUGCGGUGCCCACACUGUCAGCCCG

>M2_2R Mutant ([U25G, C28A, G37C, A38U])
UUUUUUAGCCGUAAUGAGCGUCGGGGCACACACUGUCUGCGCG

>M2_1 Mutant (Iteration 1) ([C19U, G20C, U21G, G26A, C27G, C29G, U36C, G37A])
UUUUUUAGCCGUAAUGAGUCGCGGUAGCGACACUGCAAGCGCG

>M2_1 Mutant (Iteration 2) ([C19U, G20C, U21G, C22U, G26A, C27U, C29G, U36C, U37A])
UUUUUUAGCCGUAAUGAGUCGUGGUAUCGACACUGCAAGCGCG
```

S2. KineFold helices and free energy traces

This section provides a detailed breakdown of the KineFold co-transcriptional simulation results that support the analysis presented in Section 3.5 of the main manuscript. While the main text summarizes the key events of the folding cascades, the data here offer a more granular view for full methodological transparency and reproducibility. The following subsections detail the specific definitions of the five reporter helices that were tracked for the wildtype and each mutant construct. Accompanying these descriptions, Figure S1 presents the complete free-energy traces from each simulation, illustrating the precise energetic progression of the folding pathway as each helix forms and the system settles into its final kinetic conformation.

S2.1 Wildtype FSE

The free-energy trace shows a gradual, stepwise descent from 0 to –0.28 kcal/mol over 3000 µs, with distinct drops corresponding to stem nucleation and stabilization. The RNA remains largely unstructured until $t \approx 500$ µs, when Helix 1 briefly forms, producing a transient energy drop that reverses by $t \approx 800$ µs. A more substantial and sustained descent begins at $t \approx 900$ µs, as Helices 2, 3, and 4 begin to form in coordination. These three helices persist until $t \approx 1500$ µs, after which all but Helix 4 are disrupted simultaneously, causing a temporary energy plateau. A second, more persistent round of Helices 2, 3, and 4 formation occurs beginning at $t \approx 1900$ µs and continues through the end of the simulation, gradually lowering the free energy to –0.28 kcal/mol.

S2.2 M3_6 mutant

Five helices were tracked:

- Helix 1: bases 12-24 (5 bp)
- Helix 2: bases 25-50 (4 bp)
- Helix 3: bases 14–44 (6 bp)
- Helix 4: bases 50–79 (7 bp)
- Helix 5: bases 31–81 (6 bp)

The free-energy trace shows a monotonic decrease from 0 to approximately –0.28 kcal/mol over 3000 µs. Small downward steps are visible every 200–400 µs, consistent with staged helix formation. A sharp drop at $t \approx 1200$ µs marks the nucleation of Helices 4 and 5, after which the energy steadily decreases toward the pseudoknot-locked minimum.

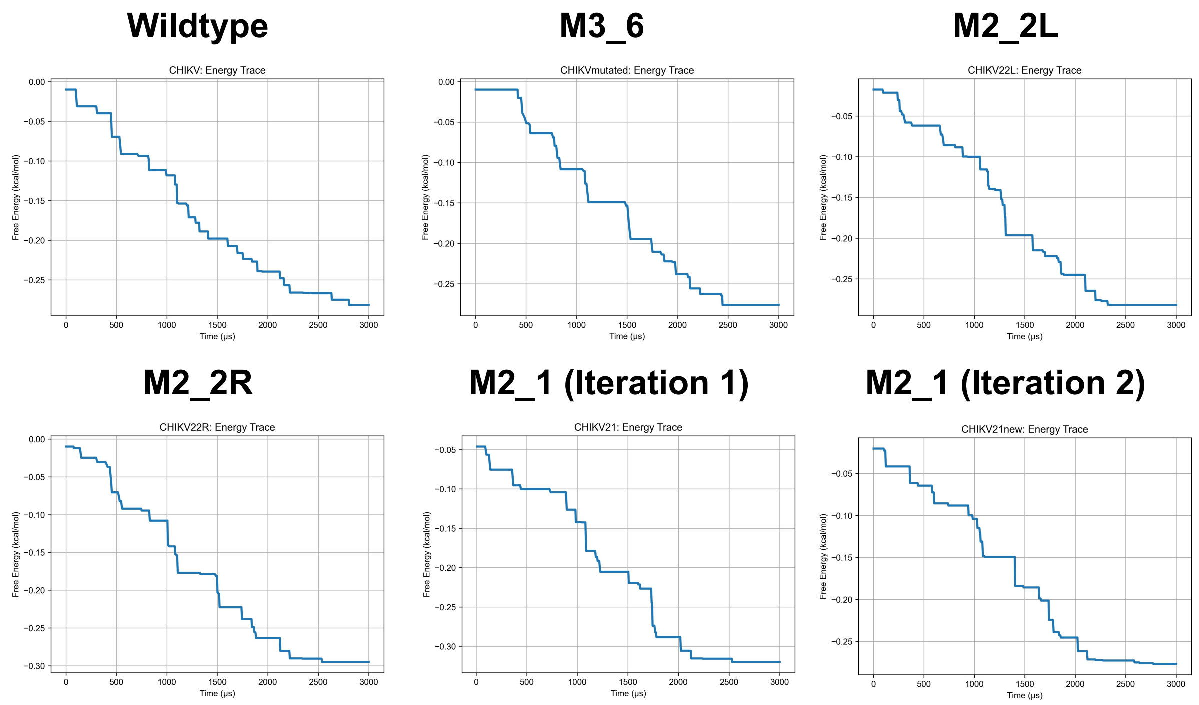

Figure S1. Co-transcriptional free energy traces for all constructs. Free energy (kcal/mol) as a function of simulation time (μs) from KineFold simulations for the wildtype FSE and all five mutant constructs (M3_6, M2_2L, M2_2R, and the two iterations for M2_1). Each downward step in a trace corresponds to a discrete folding event, such as the formation of a new helix, that lowers the overall free energy of the system. These traces illustrate the different energetic pathways each sequence takes during co-transcriptional folding.

S2.3 M2_2L mutant

Five helices were monitored:

- Helix 1: bases 20–87 (10 bp)
- Helix 2: bases 27–78 (7 bp)
- Helix 3: bases 63–79 (4 bp)
- Helix 4: bases 31–76 (6 bp)
- Helix 5: bases 77–96 (5 bp)

The free-energy trace shows a gradual decrease from 0 to approximately −0.27 kcal/mol over the 3000 μs transcription interval, with two clear inflection points. A small energy drop at $t \approx 600$ μs reflects early stem nucleation, followed by a larger, sustained descent at $t \approx 1000$ μs as the stabilized 2_2L hairpin forms and dominates the folding ensemble.

S2.4 M2_2R mutant

We tracked five helices:

- Helix 1: bases 23–121 (5 bp)
- Helix 2: bases 21–68 (6 bp)
- Helix 3: bases 20–112 (6 bp)
- Helix 4: bases 63–79 (5 bp)
- Helix 5: bases 31–76 (6 bp)

The free-energy trace shows a clean, stepwise descent from 0 to approximately −0.27 kcal/mol over the 3000 μs window, with one prominent transition at $t \approx 1000$ μs. This drop corresponds precisely to the emergence of Helix 2 (orange), which nucleates sharply at $t \approx 1000$ μs and remains stably occupied for the remainder of the simulation. No other tracked helices rise above baseline noise levels.

S2.5 M2_1 mutant, Iteration 1

The following five helices were tracked across the 43–143 nt window:

- Helix 1: bases 18–31 (5 bp)
- Helix 2: bases 21–86 (6 bp)
- Helix 3: bases 63–79 (5 bp)
- Helix 4: bases 8–75 (5 bp)
- Helix 5: bases 24–115 (5 bp)

The free-energy trace reveals multiple minor inflection points, with a gradual decline from 0 to approximately –0.32 kcal/mol. The absence of large cooperative energy drops suggests a distributed, modular folding path rather than abrupt pseudoknot formation.

S2.6 M2_1 mutant, Iteration 2

The following five helices were tracked across the 43–143 nt window:

- Helix 1: bases 20-83 (5 bp)
- Helix 2: bases 21–86 (6 bp)
- Helix 3: bases 18-31 (5 bp)
- Helix 4: bases 63-79 (5 bp)
- Helix 5: bases 8-30 (5 bp)

The free-energy trace for the redesigned M2_1 mutant shows a direct and efficient folding pathway. The simulation begins with a brief period of minimal energy change, followed by a small, initial drop around $t \approx 600$ μs as a transient hairpin forms. The defining feature of the trace is a sharp, cooperative energy drop at $t \approx 1200$ μs, where the system rapidly commits to the target 2_1 conformation. Following this key event, the energy continues to decrease in smaller, stepwise increments, indicating minor structural refinements as the system settles into a stable final state at approximately –0.28 kcal/mol.

S3. Conformational landscapes and kinetic data of M2_1 mutant, Iteration 1

To force the CHIKV frameshifting element (FSE) into the 2_1 two-stem conformation—which is also never sampled in the native energy landscape—we introduced an eight-nucleotide substitution [C19U, G20C, U21G, G26A, C27G, C29G, U36C, G37A]. These changes are specifically chosen to destabilize all competing folds (3_6, 2_3, 2_2*, 2_2L, 2_2) and to create the base-pairing pattern required for the 2_1 topology.

In every downstream sequence fragment analyzed, the wildtype FSE never adopts the 2_1 pseudoknot (0.00% probability). Upon introducing the eight mutations, the 2_1 structure becomes the dominant fold across nearly all downstream lengths. Quantitatively, the overall folding probability for the 2_1 motif rises from 0.00% in the wildtype to 68.11% in the mutant, reflecting a dramatic reorientation of the folding equilibrium toward this otherwise inaccessible conformation.

A similarly marked effect is observed in upstream sequence contexts. There, the native sequence again shows no 2_1 population (0.00%), whereas the engineered construct drives the 2_1 pseudoknot to 36.99% overall probability. In both downstream and upstream panels, all alternative structures collapse to low or zero probability, confirming that the eight-point mutation set enforces near-exclusive formation of the 2_1 pseudoknot.

While the eight-point mutation set successfully promotes 2_1 folding, requiring so many substitutions underscores the difficulty of fully suppressing alternative structures. Moreover, even with extensive sequence remodeling, the 2_1 pseudoknot never reaches complete dominance ($\geq 70\%$), illustrating the inherent challenge of driving an entirely inaccessible fold to thermodynamic prevalence.

For this first iteration, the KineFold helix occupancy plot shows a complex folding sequence (Figure 9). Helix 1 briefly appears at $t \approx 100$–450 μs, after which the ensemble enters a transiently unfolded phase. At $t \approx 1100$ μs, Helix 3 stabilizes and is joined by Helix 1 at $t \approx 1300$ μs and Helix 4 at $t \approx 1650$ μs, forming a short-lived 2_1-like ensemble. However, from $t \approx 1750$ μs onward, only Helices 2 and 3 remain persistently occupied.

The overall folding cascade can be summarized as:

1. **Initial unstructured state (0–100 μs):** RNA remains unfolded; no tracked helices are stably formed.
2. **Early stem sampling (100–450 μs):** Brief nucleation of Helix 1 leads to a transient 2_2-like conformation.
3. **Transient disruption (450–1100 μs):** The structure destabilizes, returning to an unfolded ensemble.
4. **Hairpin formation (1100–1300 μs):** Helix 3 forms and stabilizes; conformation resembles 2_2.

5. **2_1-like assembly (1300–1650 μs):** Helices 1, 3, and 4 co-occupy, producing a brief 2_1 motif window.
6. **Late-stage convergence (1650–3000 μs):** Helices 2 and 3 persist, stabilizing a 2_2R-like structure.

The folding dynamics agree only partially with equilibrium expectations. While the conformational landscape favors the 2_1 motif in a modest subregion of downstream lengths, KineFold shows only a transient 2_1-like window between 1300–1650 μs. The mutant spends the majority of the trajectory in 2_2 or 2_2R configurations. This suggests that the current eight-point mutation, while partially successful, does not fully suppress competing structures. Therefore, we concluded that additional stabilizing substitutions was required to fully enforce 2_1 adoption under cotranscriptional conditions.

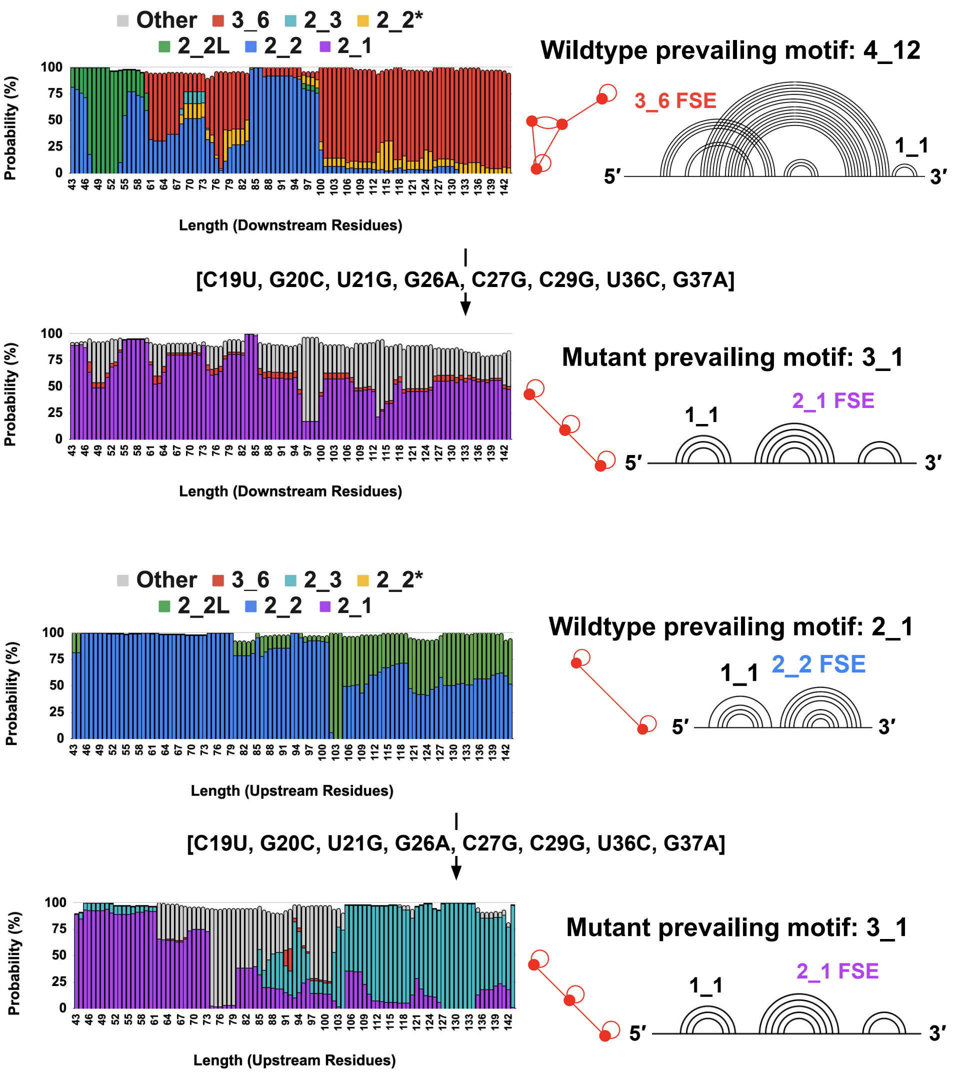

Figure S2. Thermodynamic folding landscapes of the first iteration of the M2_1 mutant. This figure compares the equilibrium conformational landscapes for the wildtype FSE (top) and the original eight-nucleotide M2_1 mutant (bottom) in both downstream (top half) and upstream (bottom half) sequence contexts. The mutations ([C19U, G20C, U21G, G26A, C27G, C29G, U36C, G37A]) successfully reconfigure the energy landscape, driving the population of the target 2_1 motif (purple) from 0% in the wildtype to 68.11% (downstream) and 36.99% (upstream). This demonstrates the thermodynamic success of the initial design, which was later shown to be kinetically frustrated (see main text, Section 3.5).
